# ACE inhibitors and angiotensin receptor blockers differentially alter the response to angiotensin II treatment in vasodilatory shock

**DOI:** 10.1186/s13054-024-04910-6

**Published:** 2024-04-18

**Authors:** Daniel E. Leisman, Damian R. Handisides, Laurence W. Busse, Mark C. Chappell, Lakhmir S. Chawla, Michael R. Filbin, Marcia B. Goldberg, Kealy R. Ham, Ashish K. Khanna, Marlies Ostermann, Michael T. McCurdy, Christopher D. Adams, Tony N. Hodges, Rinaldo Bellomo, The ATHOS-3 Investigators

**Affiliations:** 1 Department of Medicine, Massachusetts General Hospital, 55 Fruit St., GRB 7-730, Boston, MA 02114 USA; 2 Innoviva Specialty Therapeutics, Inc - an Affiliate of La Jolla Pharmaceutical Company, Waltham, MA USA; 3 Department of Medicine, Emory University, Atlanta, GA USA; 4 Emory Critical Care Center, Emory Healthcare, Atlanta, GA USA; 5 Hypertension and Vascular Research Center, Wake Forest University School of Medicine, Winston-Salem, NC USA; 6 Department of Medicine, Veterans Affairs Medical Center, San Diego, CA USA; 7 Department of Emergency Medicine, Massachusetts General Hospital, Boston, MA USA; 8 Department of Emergency Medicine, Harvard Medical School, Boston, MA USA; 9 Broad Institute of Massachusetts Institute of Technology and Harvard, Cambridge, MA USA; 10 Division of Infectious Diseases, Department of Medicine, Center for Bacterial Pathogenesis, Massachusetts General Hospital, Boston, MA USA; 11 Department of Microbiology, Harvard Medical School, Boston, MA USA; 12 Department of Critical Care, Mayo Clinic, Phoenix, AZ USA; 13 Section on Critical Care Medicine, Department of Anesthesiology, Wake Forest University School of Medicine, Atrium Health Wake Forest Baptist Medical Center, Winston-Salem, NC USA; 14 Perioperative Outcomes and Informatics Collaborative (POIC), Winston-Salem, NC USA; 15 Outcomes Research Consortium, Cleveland, OH USA; 16 Department of Critical Care, King’s College London, Guy’s & St Thomas’ Hospital, London, UK; 17 Division of Pulmonary & Critical Care Medicine, Department of Medicine, University of Maryland School of Medicine, Baltimore, MD USA; 18 Department of Emergency Medicine, University of Maryland School of Medicine, Baltimore, MD USA; 19 Australian and New Zealand Intensive Care Research Centre (ANZIC-RC), School of Public Health and Preventive Medicine, Monash University, Melbourne, Australia; 20 Department of Critical Care, Melbourne Medical School, University of Melbourne, Austin Hospital, Melbourne, Australia; 21 Data Analytics Research and Evaluation (DARE) Centre, Austin Hospital, Melbourne, Australia; 22 Department of Intensive Care Medicine, Austin Hospital, Melbourne, Australia; 23 The Australian and New Zealand Intensive Care Society (ANZICS) Centre for Outcome and Resource Evaluation (CORE), Melbourne, Australia; 24 Intensive Care Unit, Royal Melbourne Hospital, Melbourne, VIC Australia

**Keywords:** Angiotensin II, Renin–angiotensin system, Norepinephrine, Shock, Septic

## Abstract

**Background:**

Angiotensin-converting enzyme inhibitor (ACEi) and angiotensin receptor blockers (ARB) medications are widely prescribed. We sought to assess how pre-admission use of these medications might impact the response to angiotensin-II treatment during vasodilatory shock.

**Methods:**

In a *post-hoc* subgroup analysis of the randomized, placebo-controlled, Angiotensin Therapy for High Output Shock (ATHOS-3) trial, we compared patients with chronic angiotensin-converting enzyme inhibitor (ACEi) use, and patients with angiotensin receptor blocker (ARB) use, to patients without exposure to either ACEi or ARB. The primary outcome was mean arterial pressure after 1-h of treatment. Additional clinical outcomes included mean arterial pressure and norepinephrine equivalent dose requirements over time, and study-drug dose over time. Biological outcomes included baseline RAS biomarkers (renin, angiotensin-I, angiotensin-II, and angiotensin-I/angiotensin-II ratio), and the change in renin from 0 to 3 h.

**Results:**

We included n = 321 patients, of whom, 270 were ACEi and ARB-unexposed, 29 were ACEi-exposed and 22 ARB-exposed. In ACEi/ARB-unexposed patients, angiotensin-treated patients, compared to placebo, had higher hour-1 mean arterial pressure (9.1 mmHg [95% CI 7.6–10.1], *p* < 0.0001), lower norepinephrine equivalent dose over 48-h (*p* = 0.0037), and lower study-drug dose over 48-h (*p* < 0.0001). ACEi-exposed patients treated with angiotensin-II showed similarly higher hour-1 mean arterial pressure compared to ACEi/ARB-unexposed (difference in treatment-effect: − 2.2 mmHg [95% CI − 7.0–2.6], p_interaction_ = 0.38), but a greater reduction in norepinephrine equivalent dose (p_interaction_ = 0.0031) and study-drug dose (p_interaction_ < 0.0001) over 48-h. In contrast, ARB-exposed patients showed an attenuated effect of angiotensin-II on hour-1 mean arterial pressure versus ACEi/ARB-unexposed (difference in treatment-effect: − 6.0 mmHg [95% CI − 11.5 to − 0.6], p_interaction_ = 0.0299), norepinephrine equivalent dose (p_interaction_ < 0.0001), and study-drug dose (p_interaction_ = 0.0008). Baseline renin levels and angiotensin-I/angiotensin-II ratios were highest in ACEi-exposed patients. Finally, angiotensin-II treatment reduced hour-3 renin in ACEi/ARB-unexposed and ACEi-exposed patients but not in ARB-exposed patients.

**Conclusions:**

In vasodilatory shock patients, the cardiovascular and biological RAS response to angiotensin-II differed based upon prior exposure to ACEi and ARB medications. ACEi-exposure was associated with increased angiotensin II responsiveness, whereas ARB-exposure was associated with decreased responsiveness. These findings have clinical implications for patient selection and dosage of angiotensin II in vasodilatory shock.

*Trial Registration* ClinicalTrials.Gov Identifier: NCT 02338843 (Registered January 14th 2015).

**Supplementary Information:**

The online version contains supplementary material available at 10.1186/s13054-024-04910-6.

## Introduction

Vasodilatory shock, requiring vasopressor therapy, is common in ICU patients [1]. Its treatment typically relies on catecholamines, such as norepinephrine, which increase MAP by stimulating adrenergic receptors [2]. However, catecholamines, particularly at high doses, are associated with adverse effects including tachydysrhythmias, peripheral ischemia, cardiomyopathy, kidney injury, impaired cerebral perfusion, and immunosuppression [1, 3–6]. Accordingly, there is interest in agents that increase vascular tone and MAP through alternative mechanisms [5–8].

Synthetic angiotensin-II is an alternative vasopressor approved to treat hypotension in vasodilatory shock after the ATHOS-3 trial demonstrated increased MAP and decreased total vasopressor requirements compared with placebo [8]. Angiotensin-II is an endogenous octa-peptide of the renin-angiotensin system (RAS) that acts on vascular angiotensin-II type-1 receptors (AT1R) to increase systemic vascular tone. A potential challenge in using angiotensin-II to treat vasodilatory shock is that many patients chronically take RAS-inhibiting medications, such as angiotensin-converting enzyme inhibitors (ACEi) and angiotensin receptor blockers (ARB). These medications are typically withheld when patients develop shock, but have variable half-lives, residual mechanisms of action, and pharmacokinetics [9, 10]. Therefore, they could interact with angiotensin-II treatment even if ceased at hospital admission and their interaction is likely to vary according to mechanism of action.

**Fig. 1:**
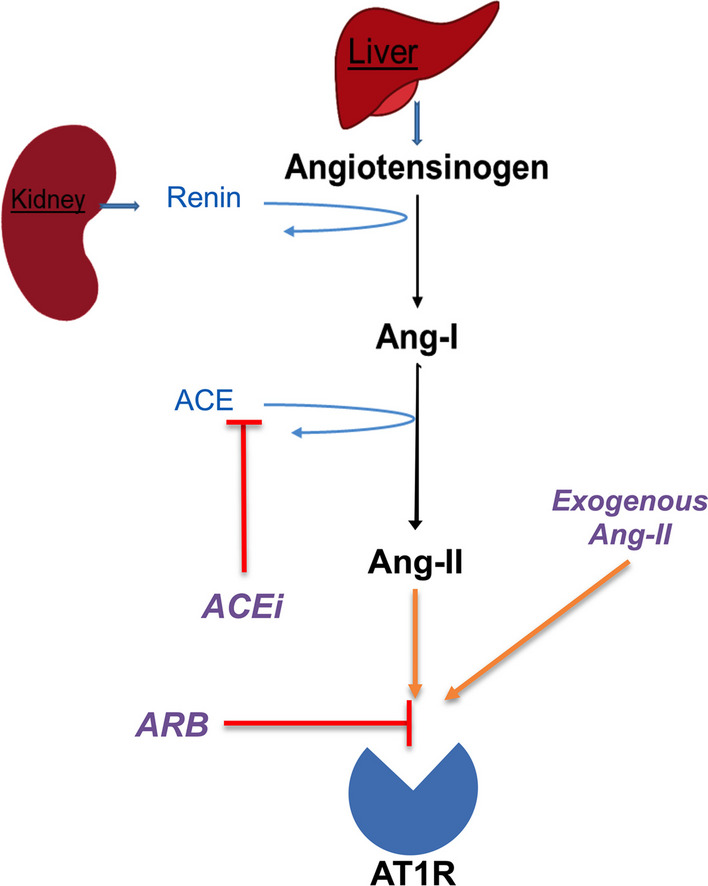
Schematic Diagram of the Classical RAS Axis and the Mechanism of ACEi and ARB medications. Renin catalyzes the conversion of angiotensinogen to angiotensin-I, which in turn is converted to angiotensin-II by ACE. Angiotensin-II primarily exerts its cardiovascular effects by stimulating the AT1R. ACEi medications act by inhibiting the conversion of the angiotensin-I to angiotensin-II. ARB medications act by directly antagonizing AT1R. *Ang* angiotensin, *ACE* angiotensin-converting enzyme, *ACEi* ACE-inhibitor, *ARB* angiotensin receptor blocker, *AT1R* angiotensin-II type-1 receptor, *RAS* renin-angiotensin system

ACEis decreases endogenous angiotensin-II generation without antagonizing AT1R and should allow a full response to angiotensin-II treatment (Fig. 1). In contrast, ARBs directly block AT1Rs, and consequently may decrease responsiveness to angiotensin-II treatment. It is also possible that ACEis might increase sensitivity to angiotensin-II through chronic upregulation of AT1R given depletion of the endogenous angiotensin-II ligand, and a possible compensatory response to restore blood pressure to hypertensive levels. Clinical evidence on how RAS-blocking therapy influences the response to angiotensin-II in shock remains limited, though the implications of such knowledge are clinically relevant.

Accordingly, we performed a post-hoc, secondary analysis of the ATHOS-3 randomized clinical trial to investigate the effect of prior ACEi and ARB exposure, respectively, on the cardiovascular and biological RAS profile response to angiotensin-II in vasodilatory shock.

## Methods

### ATHOS-3 trial

The ATHOS-3 trial has been previously described (clinicaltrials.gov identifier NCT 02338843) [8]. Briefly, adults with persistent vasodilatory shock after ≥ 25 mL/kg of volume resuscitation requiring high-dose vasopressors (i.e., norepinephrine-equivalent dose [NED] > 0.2 μg/kg/min) were randomly assigned 1:1 to receive synthetic human angiotensin-II (La Jolla Pharmaceutical Co.) or saline placebo plus standard vasopressors. Randomization was stratified by MAP at screening and Acute Physiology and Chronic Health Evaluation II (APACHE-II) score.

### Objectives

The present study reflects a post-hoc analysis of ATHOS-3 that aimed to answer the following questions:

1. Does prior exposure to ARB or ACEi therapy alter the efficacy of angiotensin-II treatment for hypotension in vasodilatory shock?
2. Does prior exposure to ARB or ACEi therapy, alter the systemic RAS biomarker profile in vasodilatory shock and the RAS response to angiotensin-II treatment?

### Exposures

The co-primary exposures of interest were exposure to ARB or ACEi therapy within the 7 days prior to randomization, which were assessed as potential effect modifiers for angiotensin-II treatment. Exposure was determined through a combination of history taken from patients or their representative and review of the electronic/patient medical record by study personnel.

As an exploratory analysis, the degree of ARB exposure was further assessed as a continuous variable by determining the equipotent dose levels in losartan equivalents (Additional file 1: Table-S1), using previously established conversion factors [10].

In the ATHOS-3 trial, study drug infusion was started at 20 ng/kg/min and titrated during hr_0_-hr_3_ to achieve MAP ≥ 75 mmHg while keeping other vasopressor doses constant. Thereafter, study drug and other vasopressors were titrated at treating clinicians’ discretion to maintain MAP between 65 and 75 mmHg. At hr_48_, study drug infusion was discontinued according to a protocol-specified tapering process but could be continued for up to 7 days per clinician discretion.

### Outcomes

The primary outcome was MAP at hr_1_. This was selected because we reasoned the immediate MAP change best reflected the hemodynamic efficacy of angiotensin-II and because using a continuous variable (rather than the binary primary endpoint of the ATHOS-3 trial) would reduce the sample size needed to statistically interrogate an interaction effect. Additionally, we compared the NED and the study-drug titration level during hr_0-3_ (i.e., during the titration period where background vasopressors were held constant), and during hr_4_-hr_48_ (i.e., the period where background vasopressors were not held constant). To investigate the association of prior ACEi and ARB exposure with the biomolecular RAS profile, we compared the baseline levels of renin, angiotensin-I, angiotensin-II, and the angiotensin-I/angiotensin-II ratio, and the change in renin at hr_3_.

Patient-centered outcomes (e.g., 28-day mortality) were not analyzed due to insufficient sample size.

### Statistical analysis

Continuous variables are reported as mean (SD) or median (interquartile range), as appropriate, and categorical variables as frequency (percent). Analyses were performed in SAS (*SAS Institute, Cary, NC, USA*.)

In the primary analysis, MAP and NED at hr_1_-hr_3_ were modeled in a linear regression that included terms for treatment and hr_0_ value of the dependent variable in accordance with best statistical practice for evaluating continuous outcomes in clinical trials [11, 12]. These models were performed when stratified by exposure status (ARB, ACEi, no ACEi/ARB), and including an interaction-term for whether exposure modified the effect of treatment.

For longitudinal modeling of NED and study drug dose, a mixed-effects repeated measures model was used with a random intercept for individual patients and unstructured covariance. The model included fixed-effects for time, treatment, ACEi and ARB exposure, as well as interaction effects between these terms to assess heterogeneity of treatment effect. Models were separately constructed for the active titration period (hr_0-3_) and the subsequent intervention period (hr_4-48_). These models have the advantage of being robust to missing data bias, even when data are not missing at random [13]. To account for right-skewed data in NED, data were log-transformed for modeling.

Differences in baseline levels of RAS biomarkers were compared with generalized linear models with post-hoc adjustment for multiple comparisons. Again, to account for right-skewed data, biomarker levels were log-transformed for analysis. To facilitate intuitive interpretation, we report back-transformed differences and least-squares means (i.e., the geometric mean [gmean]).

We used two sets of multivariable models to account for potential confounding in ACEi/ARB exposure status. The primary analysis models used a parsimonious approach, adjusted for age, sex, baseline APACHE-II score (as a measure of overall illness severity), and baseline NED (as a measure of circulatory failure severity, specifically). These covariates were selected prior to analysis as they were felt to reflect the most important sources of potential confounding. In sensitivity analyses, we also assessed the main outcomes using models that adjusted for the previous covariates as well as baseline MAP, history of chronic kidney disease, chronic hypertension, and factors that had previously been associated with response to angiotensin-II [8, 14–16]: baseline albumin, ARDS at baseline, and whether patients were on renal replacement therapy (RRT) at baseline. We used complete case-analysis as the first set of models had no missing data and the second set of models had only one covariate with missing data (albumin), which was missing in less than 5.0% of patients in all three exposure groups. We considered the use of inverse-probability of treatment weighting as an additional method of covariate adjustment, but the sample size was too small to facilitate effective balancing of the weighted population. As such we limit the presented analysis to traditional covariate adjustment.

For the exploratory analysis of ARB dose exposure, the analysis dataset was limited to ARB-exposed and ACEi/ARB-unexposed patients. The unexposed patients were considered to have a losartan-equivalent dose of 0 mg/day.

## Results

### Patient characteristics

Of 321 patients included in the primary ATHOS-3 study, 270 (84%) were not exposed to ACEi or ARB, while 29 (9%) patients had received ACEis (n = 15; 52% for angiotensin-II and n = 14; 48% for placebo treatment), and 22 (7%) patients had received ARBs (n = 11; 50% each arm). Baseline characteristics are displayed in Table 1 and Additional file 1: Table-S2. Additional file 1: Table-S3 reports the missing data prevalence.

**Table 1:** Baseline and treatment characteristics

Variable	No ACEi/ARB	ACEi	ARB	Total
N	270	29	22	321
Demographics and clinical factors				
Age (years)	62.0 (15.40)	61.6 (15.69)	67.8 (13.98)	62.3 (15.36)
Female—n (%)	103 (38.1%)	11 (36.7%)	13 (59.1%)	126 (39.3%)
Body mass index (kg/m^2^)	29.8 (8.47)	32.5 (10.92)	33.6 (8.95)	30.3 (8.80)
Ideal body weight (kg)	64.2 (11.69)	66.3 (11.24)	61.6 (9.24)	64.3 (11.51)
Cause of vasodilatory shock—n (%)				
Sepsis	218 (80.7%)	24 (80.0%)	18 (81.8%)	259 (80.7%)
Other—potentially sepsis	28 (10.4%)	1 (3.3%)	2 (9.1%)	31 (9.7%)
Other—not sepsis	24 (8.9%)	5 (16.7%)	2 (9.1%)	31 (9.7%)
Baseline APACHE II Score	28.2 (8.59)	27.1 (7.55)	26.7 (6.83)	28.0 (8.37)
Baseline albumin (g/dl)	2.3 (0.63)	2.5 (0.51)	2.2 (0.45)	2.3 (0.61)
ARDS at baseline—n (%)	70 (25.9%)	2 (6.7%)	9 (40.9%)	81 (25.2%)
Intubated at baseline—n (%)	249 (92.2%)	28 (93.3%)	19 (86.4%)	295 (91.9%)
RRT at screening—n (%)	79 (29.3%)	11 (36.7%)	8 (36.4%)	98 (30.5%)
Baseline cardiovascular status				
Mean arterial pressure (mmHg)	65.7 (5.46)	67.7 (6.23)	65.7 (2.95)	65.9 (5.42)
Baseline NED (µg/kg/min)	0.47 (0.412)	0.44 (0.304)	0.43 (0.378)	0.46 (0.400)
Average NED in past 6 h (µg/kg/min)	0.51 (0.399)	0.53 (0.286)	0.49 (0.301)	0.51 (0.383)
Vasopressin use in past 6 h—n (%)	188 (69.6%)	20 (66.7%)	17 (77.3%)	224 (69.8%)
Central venous pressure (mmHg)	13.5 (5.01)	12.1 (2.72)	11.9 (5.31)	13.3 (4.88)
Cardiac index (L/min/m^2^) ScvO_2_ (%)	3.3 (0.93)	3.6 (1.35)	3.6 (1.12)	3.4 (0.97)
Medical history				
Hypertension—n (%)	139 (51.5%)	23 (76.7%)	22 (100.0%)	183 (57.0%)
Chronic kidney Disease—n (%)	66 (24.4%)	11 (36.7%)	7 (31.8%)	84 (26.2%)
Diabetes—n (%)	90 (33.3%)	14 (46.7%)	9 (40.9%)	112 (34.9%)
Coronary artery disease—n (%)	70 (25.9%)	10 (33.3%)	3 (13.6%)	83 (25.9%)
Chronic Heart Failure—n (%)	49 (18.1%)	8 (26.7%)	2 (9.1%)	59 (18.4%)
Baseline lab values				
WBC (10^9^/L)—Med [IQR]	16.7 [10.0, 25.3]	19.2 [13.8, 27.4]	17.0 [12.4, 22.5]	17.2 [10.6, 25.6]
Hemoglobin (g/dL)— Med [IQR]	9.8 [8.5, 11.2]	9.3 [7.9, 11.7]	10.2 [9.1, 11.0]	9.8 [8.5, 11.2]
Potassium (mEq/L)—Med [IQR]	4.2 [3.8, 4.8]	4.4 [3.9, 4.7]	4.5 [3.9, 4.9]	4.2 [3.8, 4.8]
Creatinine (mg/dL)— Med [IQR]	1.9 [1.2, 2.8]	2.4 [1.4, 3.4]	3.0 [1.8, 4.0]	2.1 [1.2, 2.9]
BUN (mg/dL)—Med [IQR]	25 [15, 43]	33 [13, 44]	22 [17, 30]	25 [15, 43]
Bicarbonate (mEq/L)—Med [IQR]	20 [15, 23]	19.0 [15, 21]	18 [16, 20]	19 [15, 22]
P/F Ratio (mmHg)—Med [IQR]	210 [138, 288]	261 [186, 318]	212 [114, 280]	211 [140, 296]
Treatment characteristics				
Duration of study drug exposure (hr)—Med [IQR]	48.1 [35.5, 49.3]	48.0 [28.5, 48.4]	42.7 [29.1, 48.0]	48.0 [35.0, 49.2]
Fluid administration (mL)—Med [IQR]	521 [319, 789]	497 [257, 913]	537 [433, 664]	525 [319, 784]

Baseline characteristics of the cohort. Data are reported as mean (SD) unless otherwise indicated

*ARB* angiotensin receptor blocker, *ACEi* angiotensin-converting enzyme inhibitor, *APACHE* acute physiology and chronic illness evaluation score, *ARDS* acute respiratory distress syndrome, *RRT* renal replacement therapy, *NED* norepinephrine equivalent dose, *ScvO2* central venous oxygen saturation, *WBC* white blood cell count, *BUN* blood urea nitrogen, *P/F Ratio* ratio of arterial oxygen tension to fraction of inspired oxygen

### Early cardiovascular endpoints

**Fig. 2:**
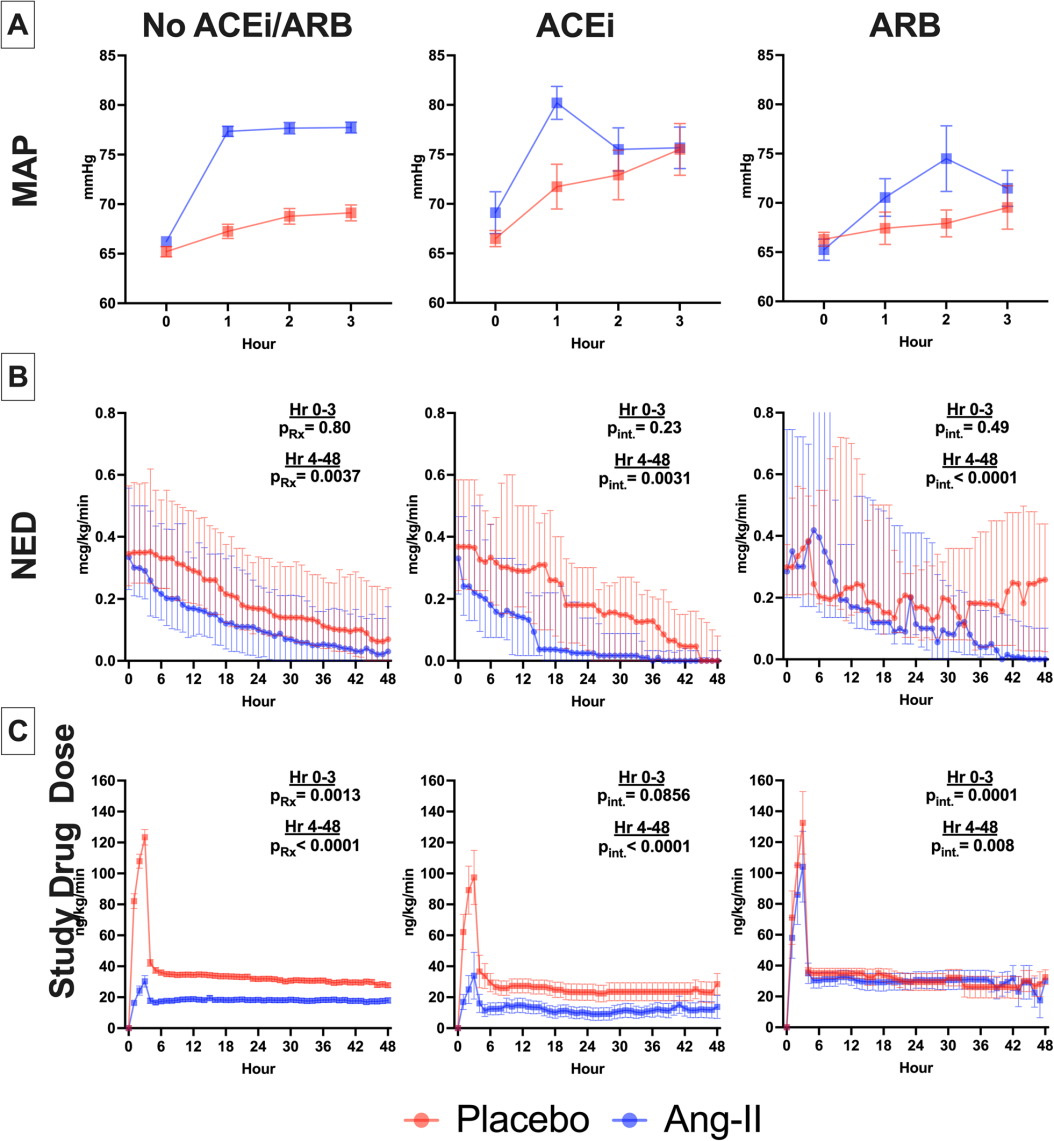
Early Cardiovascular Endpoints for Angiotensin-II versus Placebo Treatment According to ACEi and ARB Exposure Status. **A** MAP from baseline to hour 3 in Angiotensin-II (blue) versus placebo (red) treated patients, stratified by ACEi and ARB exposure status. Boxes indicate mean, error bars SEM. **B** Total NED from baseline to hour 48. Points indicate median, bars interquartile range. For No ACEi/ARB (left), p_Rx_ indicates the p-value for the overall effect of Angiotensin-II treatment versus placebo. For ACEi (middle) and ARB (right), p_int_ indicates the *p*-value for highest order interaction effect of ACEi or ARB with time and treatment, where *p* < 0.05 indicates an effect that significantly differs from the effect of treatment over time in the no ACEi/ARB group. **C** Study drug titration level over time. Boxes indicate mean, error bars SEM. *P*-values reflect longitudinal mixed effect model output as in B. *Ang-II* angiotensin-II, *ACEi* angiotensin converting enzyme inhibitor, *ARB* angiotensin receptor blocker, *MAP* mean arterial pressure, *NED* norepinephrine equivalent dose

Figure 2 displays cardiovascular measures over time. Among ACEi/ARB-unexposed patients at hr_1_, MAP increased by 11.1 mmHg [95% CI 10.0–12.3] (*p* < 0.001) with angiotensin-II, from 66 to 77 mmHg versus 2.1 mmHg from 65 to 67 mmHg [95% CI 0.9–3.1] (*p* < 0.001) in placebo-treated patients (adjusted treatment-effect: 9.1 mmHg [95% CI 7.6–10.1], *p* < 0.0001) (Fig. 2A, Additional file 1: Table-S4).

A similar increase was seen among ACEi-exposed patients, from 69 to 80 mmHg in the angiotensin-II group (difference:11.1 mmHg [95% CI 7.2–14.9], *p* < 0.0001) versus from 66 to 72 mmHg (difference: 5.8 mmHg [95% CI 1.9–9.7], *p* = 0.006) in the placebo group (adjusted difference in treatment-effect versus ACEi/ARB-unexposed: − 2.2 mmHg [95% CI − 7.0–2.6], p_interaction_ = 0.38).

In contrast, ARB exposed patients showed an attenuated increase in MAP, from 65 to 71 mmHg at hr_1_ in angiotensin-treated patients (difference: 5.3 mmHg [95% CI 1.3–9.3]; *p* = 0.0143) versus from 66 to 68 mmHg (difference: 2.2 mmHg [95% CI − 1.7–6.1]; *p* = 0.24) in placebo patients (adjusted difference in treatment-effect versus ACEi/ARB-unexposed: − 6.0 mmHg [95% CI − 11.5 to − 0.6], p_interaction_ = 0.0299). In summary, there was no difference between ACEi/ARB-unexposed and ACEi-exposed patients in the degree of MAP increase at hr_1_ with angiotensin-II versus placebo, but in ARB-exposed patients the effect of angiotensin-II to increase MAP at hr_1_ was attenuated.

The average decrease in NED from baseline during hr_0_-hr_3_ was greater for angiotensin-II compared to placebo among both ACEi/ARB-unexposed (between-group difference: 0.04mcg/kg/min [95% CI 0.02–0.06], *p* < 0.001) and ACEi-exposed patients (between-group difference: 0.04 mcg/kg/min [95% CI 0.01–0.07], *p* = 0.012). These differences reflected decreased NED in the treatment group (Fig. 2B). For ARB-exposed patients there was a nominally similar difference for angiotensin-II versus placebo (between-group difference: 0.04 mcg/kg/min [95% CI − 0.01–0.09], *p* = 0.14). However, in contrast to the ACEi/ARB-unexposed and ACEi-exposed patients, this reflected increased NED in the placebo group rather than decreased NED in the angiotensin-II group. Similar results were obtained when NED was modeled longitudinally (Fig. 2B, Additional file 1: Table-S5/S6). That is, in unexposed patients, angiotensin-II treatment reduced NED over hr_4_-hr_48_ (*p* = 0.0037). Over the hr_4_-hr_48_ period, in ACEi patients, the effect of angiotensin-II to reduce NED was enhanced compared with ACEi/ARB-unexposed patients (p_interaction_ = 0.0031). In contrast, this effect was attenuated in ARB patients (p_interaction_ < 0.0001).

The mean study-drug titration level during hr_0_-hr_3_ was lower in the angiotensin-II versus placebo groups among ACEi/ARB-unexposed patients (20.7 ng/kg/min versus 86.9 ng/kg/min; difference: to − 66.2 ng/kg/min [95% CI − 74.9 to − 57.5]; *p* < 0.001). Among ACEi-exposed patients, the angiotensin-II group had a similarly lower study-drug dose versus placebo: 22.1 ng/kg/min versus 65.3 ng/kg/min (difference: − 43.2 ng/kg/min [95% CI − 70.6 to − 15.8]; *p* = 0.003). In contrast, among ARB-exposed patients, the mean study-drug titration level during hr_0_-hr_3_ was higher and no different between angiotensin-II and placebo groups: 68.0 ng/kg/min versus 68.6 ng/kg/min (difference: − 0.6 ng/kg/min [95% CI to − 44.9–43.6]; *p* = 0.98). The longitudinal models produced similar results suggesting heterogeneity of treatment effect for angiotensin-II based on RASi exposure status (Fig. 2C, Additional file 1: Table-S7/S8).

### Biomarker endpoints

**Fig. 3:**
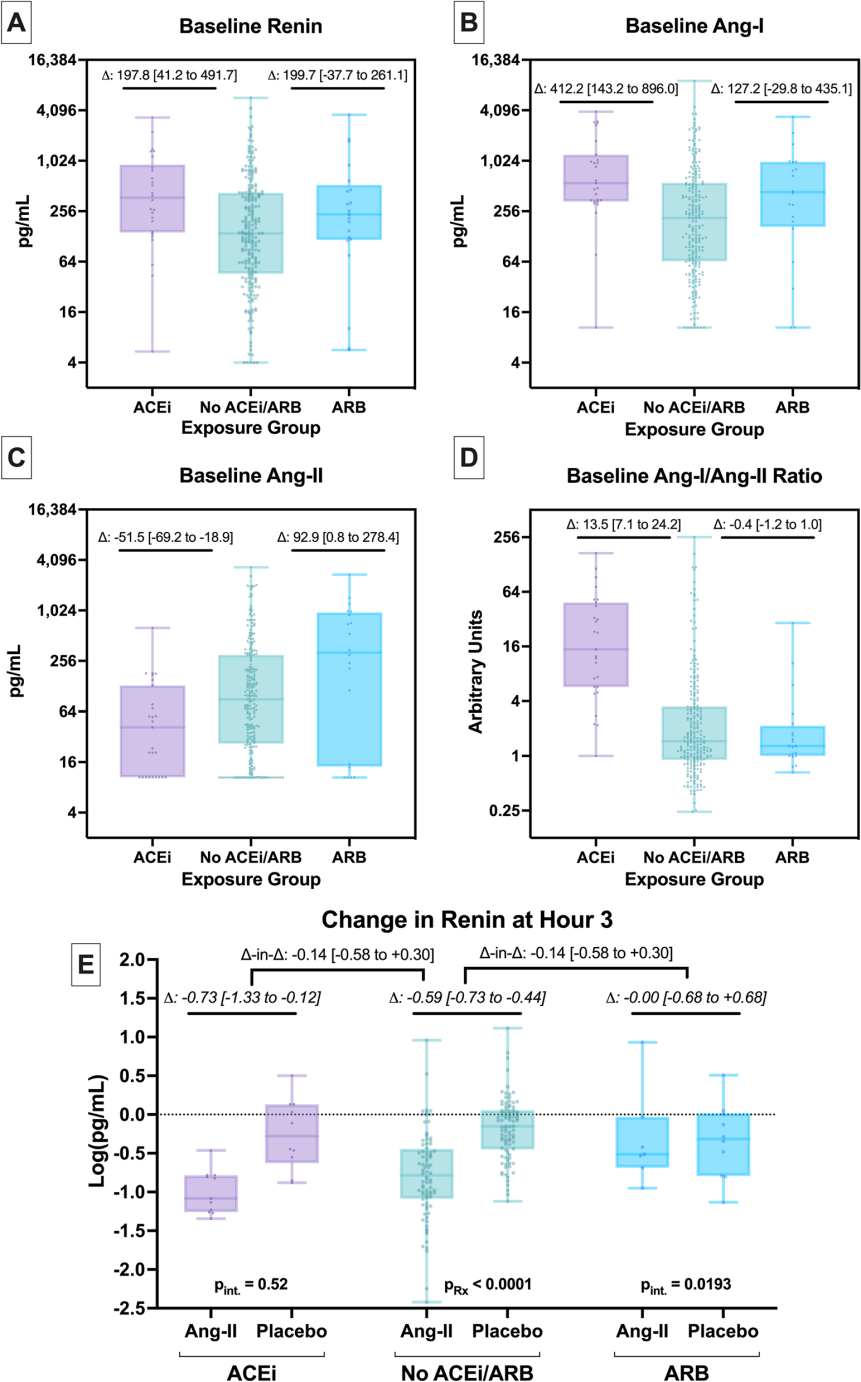
Biomolecular RAS profile according to ACEi and ARB exposure status. **A**–**D** Baseline renin, Ang-I, Ang-II, and Ang-I/Ang-II ratio according to ACEi and ARB medication exposure. Y-axes are on log2-scale. Bars indicate median, boxes IQR, whiskers data range. Dots represent individual patients. Δ indicates the difference in geometric mean between the indicated group, bracketed values the 95% CI. **E** Change in log-renin at hour 3 in angiotensin-II versus placebo treated patients, stratified by ACEi and ARB exposure status. Lower-level Δ and bracketed values reflect the difference and 95% CI from the unadjusted linear model, as above, for angiotensin-II versus placebo treatment within the ACEi/ARB exposure group. Upper-level Δ and bracketed values reflect the same, but for the interaction effect of treatment with ACEi/ARB medication group. The p_Rx_ shows the *p*-value for the effect of angiotensin-II versus placebo treatment in the no ACEi/ARB group. The p_int._ reflects the *p*-value for the interaction effect of ACEi or ARB exposure with angiotensin-II treatment, where p_int._ < 0.05 indicates a significantly different effect of treatment versus ACEi/ARB unexposed patients. *ang* angiotensin, *ACEi* angiotensin-converting enzyme inhibitor, *ARB* angiotensin receptor blocker

RAS biomarkers according to ACEi and ARB exposure are shown in Fig. 3. At baseline, renin was higher in ACEi-exposed patients (gmean: 335.3 pg/mL) than unexposed patients (gmean: 137.5 pg/mL) (difference: 197.8 pg/mL [41.2–491.7], *p* = 0.0057) (Fig. 3A). The difference was preserved after multivariable adjustment (adjusted-difference: 605.1 pg/mL [167.0–1394.1], *p* = 0.0016) (Additional file 1: Table-S9). In contrast, for ARB-exposed patients, renin was numerically higher (gmean: 199.7 pg/mL; difference: 62.2 pg/mL [− 37.7–262.1], *p* = 0.29). On multivariable adjustment, ARB-exposure was associated with higher baseline renin (adjusted-difference: 394.0 pg/mL [21.0–1113.9], *p* = 0.0341). Moreover, compared to ACEi/ARB-unexposed patients, ACEi patients had higher baseline angiotensin-I (difference: 412.2 pg/mL [143.2–896.0], *p* = 0.0002) whereas ARB patients had a nominally higher angiotensin-I (difference: 127.2 pg/mL [− 29.8–435.1], *p* = 0.14) (Fig. 3B). Conversely, baseline angiotensin-II was lower for ACEi (difference: − 51.5 pg/mL [− 69.2 to − 18.9], *p* = 0.0068) and higher for ARB (difference: 92.9 pg/mL [0.8–278.4], *p* = 0.0473) versus ACEi/ARB-unexposed patients (Fig. 3C). Finally, the angiotensin-I/angiotensin-II ratio was higher for ACEi patients (difference: 13.5 arbitrary units [7.1–24.2], *p* < 0.0001), but similar between ARB and ACEi/ARB-unexposed patients (difference: − 0.4 arbitrary units [− 1.2–1.0], *p* = 0.51) (Fig. 3D). Group differences in angiotensin-I and angiotensin-II and their ratio were similar to unadjusted results after multivariable adjustment (Additional file 1: Table-S10-S12).

At hr_3_, in both unadjusted and adjusted analysis, compared with placebo, angiotensin-II treatment decreased renin levels (*p* < 0.0001) (Fig. 3E). This effect was seen among ACEi/ARB-unexposed patients (reference group) and ACEi-exposed patients (p_interaction_ = 0.51 versus unexposed). In contrast, angiotensin-II treatment had no effect on hr_3_ renin levels among ARB-exposed patients (p_interaction_ = 0.0193 versus unexposed) such that renin levels did not decrease at hr_3_ (Additional file 1: Table-S13).

### Exploratory analyses of ARB dose

**Fig. 4:**
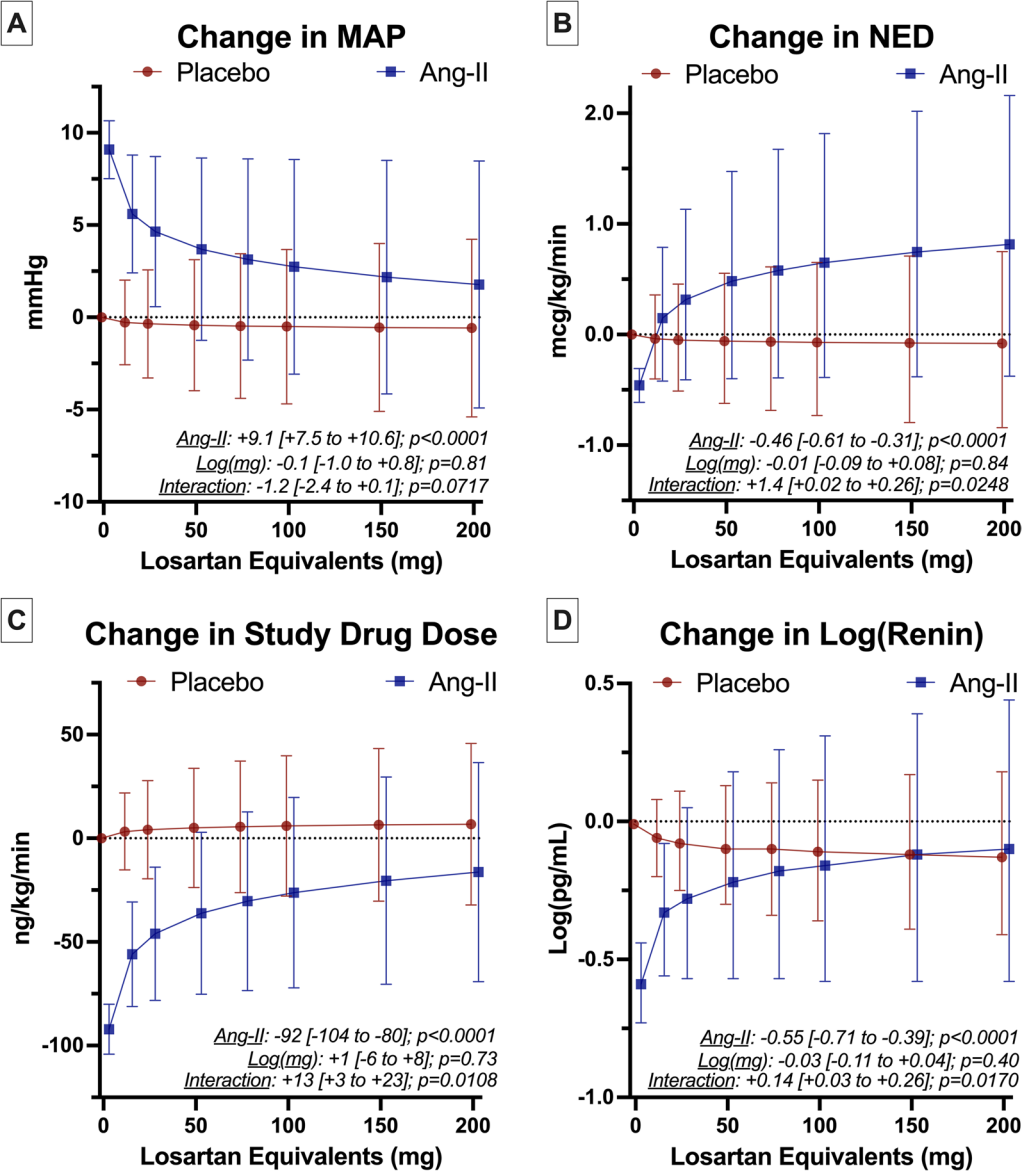
Representative Plots of Interaction between ARB dose and Effect of Angiotensin-II on Main Study Outcomes. **A**–**D** Displays marginal effect sizes from multivariable models for key study outcomes in angiotensin-II and placebo arms at clinically relevant ARB doses in losartan equivalents. The models excluded the n = 29 ACEi-exposed patients (total sample n = 292). Losartan equivalents were modeled on a natural log scale to accommodate observed distributions. Unexposed patients were considered to have an ARB dose = 0 Log(mg). To facilitate interpretation, the estimates were plotted for the corresponding untransformed values at clinically relevant ARB doses along the X-axes. Results are plotted at representative time points for MAP (hr1), NED (hr6), study drug dose (hr3), and change in renin (hr3). Circles/squares show point estimates for placebo/angiotensin-II arms, respectively. Error bars 95% CIs. Key effect estimates from the multivariable models are displayed on the graph as follows: Ang-II = the treatment effect (i.e., in ARB-unexposed patients), Log(mg) = the main-effect of losartan equivalents (i.e., the effect in the placebo group), and Interaction = the interaction-effect of losartan equivalents with treatment (i.e., change in treatment effect per log(mg) increase in ARB dose). Brackets show the 95% confidence intervals for the effects. Full model output, including main and interaction effect sizes, and their confidence intervals can be found in Additional file 1: Tables S4–S8. *Ang-II* angiotensin-II, *ACEi* angiotensin converting enzyme inhibitor, *ARB* angiotensin receptor blocker, *MAP* mean arterial pressure, *NED* norepinephrine equivalent dose

To explore whether the association of ARB exposure with attenuated response to angiotensin-II was a dose-dependent or mechanistic phenomenon, we assessed the effect of ARB dose on key outcomes. These results are summarized in Fig. 4D. Among placebo patients ARB dose was not associated with MAP at hr_1_ but among angiotensin-treated patients, each log-mg increase in losartan equivalents was associated with a 1.2 mmHg [95% CI − 2.4–0.1] (p_interaction_ = 0.0717) decrease in MAP (Additional file 1: Table-s14). Increasing losartan equivalent dose was similarly associated with higher NED and study drug dose among angiotensin-treated patients (Fig. 4B, C, (Additional file 1: Table-S15/S16). Higher ARB dose attenuated the decrease in renin at hr_3_ associated with angiotensin-treatment (difference-in-difference: 1.4 log(pg/mL)-per-log(mg) [0.02–0.26]; p_interaction_ = 0.0248) (Fig. 4D) (Additional file 1: Table-S17); i.e., renin decreased less in response to angiotensin-II therapy with increasing dose of ARB exposure.

## Discussion

### Key findings

In this post-hoc analysis of a phase-III placebo-controlled randomized clinical trial of angiotensin-II in patients with catecholamine-refractory vasodilatory shock, prior exposure to ACEi increased the cardiovascular response to angiotensin-II. In contrast, exposure to ARBs reduced such responsiveness. Moreover, after adjusting for baseline illness severity, both ACEi and ARB exposure were associated with higher baseline renin levels, but whereas both ACEi-exposed and ACEi/ARB-unexposed patients showed a renin response to angiotensin-II therapy, angiotensin-II did not reduce plasma renin levels in ARB-exposed patients. Finally, in exploratory dose–response analysis, we observed exposure to higher doses of ARB medications was associated with greater attenuation of the effect of angiotensin-II treatment versus placebo.

### Relationship to prior literature

Human synthetic angiotensin-II is a relatively new vasopressor, and identifying patients most likely to benefit from therapy is an area of active investigation. ACEi and ARB medications are prescribed to more than 15% of all U.S. adults [17], yet available data on the effect of these medications on the response to angiotensin-II treatment are limited. We note the total prevalence of pre-admission ACEi and ARB receipt in this study was 15.3%, similar to the general population prevalence. Consistent with our findings, the ARAMIS-1 study found that ARB-exposed patients had higher angiotensin-II requirements to achieve target MAP as well as higher baseline renin [18]. This study was limited by small sample size and had too few patients to assess the effect of ACEi exposure. Gleeson et al. 19] did not find an association between prior ACEi or ARB exposure and renin level but only 4 subjects were exposed to a RAS-blocking medication. Secondary analysis of the SEPSISPAM trial showed ARB-exposed, but not ACEi-exposed patients, had lower occurrence of severe AKI with higher MAP targets, which could also be congruent with our findings [20]. To our knowledge, no study has yet assessed whether the dose of ARB exposure is associated with shock outcomes or response to angiotensin-II.

### Implications of findings

Our observations imply that ACEi and ARB medications have different effects on the baseline RAS profile of patients with catecholamine-refractory vasodilatory shock. As expected, ACEi-exposed patients had greater baseline elevations in renin, angiotensin-I, and angiotensin-I/angiotensin-II ratio, and lower levels of angiotensin-II than unexposed patients. Conversely, ARB-exposed patients had higher baseline angiotensin-II, but no difference in the angiotensin-I/angiotensin-II ratio. These biomarker differences are relevant not only for understanding patient biology in vasodilatory shock, but also because several studies have found strong correlation between plasma renin level and clinical outcomes including mortality [16, 19, 21–24].

Moreover, our findings imply that ACEi and ARB exposure had opposite, yet physiologically logical (Fig. 1), effects on the cardiovascular and RAS response to angiotensin-II treatment. ACEi recipients were sensitive to angiotensin-II treatment with respect to their cardiovascular response, while ARB patients were not. Furthermore, whereas renin decreased by hr_3_ with angiotensin-II treatment versus placebo among both ACEi-exposed and ACEi/ARB-unexposed patients, neither angiotensin-II nor placebo decreased renin levels among ARB-exposed patients. These concordant clinical and biological findings are consistent with the known differences in the mechanisms of these two medication classes. Whereas ACEis inhibit angiotensin-II generation, an upstream step from exogenous angiotensin-II infusion that should not affect treatment response, ARBs antagonize AT1R which would be expected to interfere with the mechanism of action for angiotensin-II infusion. The finding that ARB exposure was dose-dependently associated with angiotensin-II treatment response may loan further credibility to the mechanistic results in this study. Given the widespread use of both medication classes, these results may have direct implications for both clinical use of angiotensin-II and future studies of angiotensin-II in shock.

### Limitations

We acknowledge several limitations. First, only 15% of ATHOS-3 patients had prior exposure to a RAS-blocking therapy. While our findings represent, to our knowledge, the largest study of angiotensin-II therapy in patients with RASi exposure, the sample size is still small. In particular, this analysis was not powered for patient-centered clinical endpoints. Second, as a post-hoc analysis, this study cannot confirm causality, particularly given that the primary exposure of interest was not randomly allocated. Third, fluid balance, filling pressures, and serial echocardiography were not measured, which could impact treatment response. However, this study focused on the early drug-titration period, during which time administered fluid volumes were captured and were not substantively different. Fourth, we did not capture the timing of the last administration date for ACEi or ARB medications, and the retained biological activity of these drugs is an assumption. However, chronic exposure to both medications classes can durably alter the RAS—e.g., telimesartan exposure can downregulate AT1R expression [25], while chronic ACEi exposure can induce an “angiotensin escape” phenomenon, likely in the setting of upregulation of ACE or of non-canonical angiotensin-II generating enzymes [26]. Fifth, the biomarkers assessed in this study primarily reflect the classical (ACE/Angiotensin-II/AT1R) axis of the RAS, but the non-classical arms of the RAS could also influence the interaction of prior RAS-inhibition and the response to angiotensin-II. Sixth, we did not formally adjust for multiple comparisons that accounted for the number of outcomes assessed, which could increase the risk of type-I error. However, this possibility is made less likely by the consistency of results across a variety of outcomes assessed and model specifications.

### Conclusion

Prior exposure to RAS-inhibiting medications was associated with an altered RAS profile and cardiovascular response to angiotensin-II treatment in patients with catecholamine-refractory vasodilatory shock. ACE-inhibitor exposure was associated with greater sensitivity to angiotensin-II treatment, whereas ARB exposure was associated with a blunted response to angiotensin-II. These findings may have clinical implications and indicate that these medications cannot be considered equivalent when initiating angiotensin-II treatment in vasodilatory shock.

### Supplementary Information

**Additional file 1.** Supplementary tables.

## Data Availability

The data that support the findings of this study were used under license from La Jolla Pharmaceutical Company for the current study. Data are available from the authors upon reasonable request and with permission of La Jolla Pharmaceutical Company.

## Abbreviations

ACEi: Angiotensin-converting enzyme inhibitor
APACHE-II: Acute physiology and chronic health evaluation II score
ARB: Angiotensin receptor blocker
AT1R: Angiotensin type-1 receptor
ATHOS-3: Angiotensin II for the treatment of high output shock trial
Gmean: Geometric mean
ICU: Intensive Care Unit
MAP: Mean arterial pressure
NED: Norepinephrine equivalent dose
RAS: Renin–angiotensin system
RASi: Renin–angiotensin system inhibitor
SEPSISPAM: Sepsis and mean arterial pressure trial
