# Supplementary material for: ACE inhibitors and angiotensin receptor blockers differentially alter the response to angiotensin II treatment in vasodilatory shock
Source: Crit Care. 2024 Apr 18;28:130. doi: 10.1186/s13054-024-04910-6 (PMC11027368; doi:10.1186/s13054-024-04910-6)
Supplement: Supplementary file 1 — Additional file 1. Supplementary tables. [file 13054_2024_4910_MOESM1_ESM.docx]

**SUPPLEMENTAL MATERIAL**

Title: ACE Inhibitor and Angiotensin Receptor Blocker Exposure Differentially Alter the Response to Angiotensin II Treatment in Vasodilatory Shock

| **Table of Contents** | |
| --- | --- |
| **Table-S1:** Dose Conversions for ARB Medications | pg. 1 |
| **Table-S2:** Baseline and Treatment Characteristics Stratified by Treatment Assignment | pg. 2 |
| **Table-S3:** Prevalence of Missing Data | pg. 3 |
| **Table-S4:** Multivariable Model for MAP at Hour-1 by ACEi/ARB Group and Treatment | pg. 4 |
| **Table-S5:** Hour 0-3 Multivariable Longitudinal Model for Effect of Treatment on NED by ACEi/ARB Exposure | pg. 5 |
| **Table-S6:** Hour 4-48 Multivariable Longitudinal Model for Effect of Treatment on NED by ACEi/ARB Exposure | pg. 6 |
| **Table-S7:** Hour 0-3 Multivariable Longitudinal Model for Effect of Treatment on Study Drug Dose by ACEi/ARB Exposure | pg. 7 |
| **Table-S8:** Hour 4-48 Multivariable Longitudinal Model for Effect of Treatment on Study Drug Dose by ACEi/ARB Exposure | pg. 8 |
| **Table-S9:** Baseline Renin as Function of ACEi/ARB Group | pg. 9 |
| **Table-S10:** Baseline Ang-I as Function of ACEi/ARB Group | pg. 10 |
| **Table-S11:** Baseline Ang-II as Function of ACEi/ARB Group | pg. 10 |
| **Table-S12:** Baseline Ang-I/Ang-II Ratio as Function of ACEi/ARB Group | pg. 11 |
| **Table-S13:** Change in Log-Renin as Function of ACEi/ARB Group and Ang-II Treatment | pg. 12 |
| **Table-S14:** Change in Hour-1 MAP by ARB Dose Equivalents and Treatment | pg. 13 |
| **Table-S15:** Change in Hour-6 NED by ARB Dose Equivalents and Treatment | pg. 14 |
| **Table-S16:** Change in Hour-3 Study Drug Dose by ARB Dose Equivalents and Treatment | pg. 15 |
| **Table-S17:** Change in Log-Renin as Function of ARB Dose Equivalents and Ang-II Treatment | pg. 16 |

| **Table-S1: Dose Conversions for ARB Medications** | | |
| --- | --- | --- |
| Drug | Dose Equivalence (mg) | Conversion Factor |
| Losartan | 50 | 1.000 |
| Candesartan | 8 | 6.250 |
| Valsartan | 80 | 0.625 |
| Olmesartan | 10 | 5.000 |
| Telmisartan | 20 | 2.500 |
| Irbesartan | 75 | 0.667 |
| Displays the equivalent doses of ARB medications that study patients were exposed to. Doses reflect the total daily dose. The conversion factor reflects the coefficient to convert the dose of the indicated medication into mg of losartan. | | |

| **Table-S2: Baseline and Treatment Characteristics Stratified by Treatment Assignment** | | | | | | |
| --- | --- | --- | --- | --- | --- | --- |
|  | **No ACEi or ARB** | | **ACEi** | | **ARB** | |
| **Variable** | Placebo | Ang-II | Placebo | Ang-II | Placebo | Ang-II |
| **N** | 133 (49.3%) | 137 (50.7%) | 14 (48.3%) | 15 (51.7%) | 11 (50.0%) | 11 (50.0%) |
| **Demographics and Clinical Factors** | | | | | | |
| Age (years) | 62.0 (15.03) | 62.0 (15.81) | 63.0 (16.13) | 60.3 (15.68) | 69.9 (15.09) | 65.6 (13.14) |
| Female – n (%) | 43 (32.3%) | 60 (43.8%) | 7 (46.7%) | 4 (26.7%) | 6 (54.5%) | 7 (63.6%) |
| Body Mass Index (kg/m^2^) | 30.3 (8.82) | 29.3 (8.12) | 34.4 (11.33) | 30.8 (10.61) | 34.4 (10.88) | 33.0 (7.25) |
| Ideal Body Weight (kg) | 65.3 (10.94) | 63.2 (12.33) | 67.5 (13.06) | 65.1 (9.57) | 62.3 (8.86) | 60.9 (9.96) |
| Cause of Shock – n (%) |  |  |  |  |  |  |
| Sepsis | 112 (84.2%) | 106 (77.4%) | 13 (86.7%) | 11 (73.3%) | 8 (72.7%) | 10 (90.9%) |
| Other – Potentially Sepsis | 10 (7.5%) | 18 (13.1%) | 0 (0.0%) | 1 (6.7%) | 1 (9.1%) | 1 (9.1%) |
| Other – Not Sepsis | 11 (8.3%) | 13 (9.5%) | 2 (13.3%) | 3 (20.0%) | 2 (18.2%) | 0 (0.0%) |
| Baseline APACHE II Score | 29.1 (8.43) | 27.3 (8.69) | 26.5 (7.38) | 27.7 (7.94) | 26.5 (8.05) | 26.8 (5.76) |
| Baseline Albumin (g/dl) | 2.3 (0.57) | 2.2 (0.68) | 2.5 (0.59) | 2.5 (0.44) | 2.2 (0.50) | 2.1 (0.42) |
| ARDS at Baseline – n(%) | 42 (31.6%) | 28 (20.4%) | 2 (13.3%) | 0 (0.0%) | 3 (27.3%) | 6 (54.5%) |
| Intubated at Baseline – n(%) | 125 (94.0%) | 124 (90.5%) | 14 (93.3%) | 14 (93.3%) | 9 (81.8%) | 10 (90.9%) |
| RRT at Screening – n(%) | 47 (35.3%) | 32 (23.4%) | 6 (40.0%) | 5 (33.3%) | 3 (27.3%) | 5 (45.5%) |
| **Baseline Cardiovascular Status** | | | | | | |
| MAP (mmHg) | 65.2 (5.95) | 66.2 (4.90) | 66.3 (3.03) | 69.1 (8.19) | 66.1 (2.26) | 65.2 (3.57) |
| Average NED in past 6 hours (µg/kg/min) | 0.53 (0.444) | 0.48 (0.349) | 0.64 (0.325) | 0.42 (0.201) | 0.38 (0.151) | 0.59 (0.379) |
| Vasopressin use in past 6 hours – n (%) | 94 (70.7%) | 94 (68.6%) | 11 (73.3%) | 9 (60.0%) | 7 (63.6%) | 10 (90.9%) |
| CVP (mmHg) | 13.0 (4.72) | 13.9 (5.25) | 11.8 (2.32) | 12.4 (3.10) | 11.4 (5.76) | 13.0 (4.69) |
| Cardiac Index (L/min/m^2^) | 3.4 (1.02) | 3.2 (0.79) | 3.4 (1.14) | 3.9 (1.61) | 3.8 (1.34) | 3.5 (1.03) |
| ScvO_2_ (%) | 77.6 (7.88) | 77.4 (8.69) | 77.0 (8.07) | 78.4 (11.07) | 74.1 (14.46) | 79.2 (9.86) |
| **Medical History** | | | | | | |
| Hypertension – n(%) | 63 (47.4%) | 76 (55.5%) | 13 (86.7%) | 10 (66.7%) | 11 (100.0%) | 11 (100.0%) |
| CKD – n(%) | 40 (30.1%) | 26 (19.0%) | 6 (40.0%) | 5 (33.3%) | 4 (36.4%) | 3 (27.3%) |
| Diabetes – n (%) | 48 (36.1%) | 42 (0.7%) | 8 (53.3%) | 6 (40.0%) | 6 (54.5%) | 3 (27.3%) |
| CAD – n(%) | 30 (22.6%) | 40 (29.2%) | 4 (26.7%) | 6 (40.0%) | 1 (9.1%) | 2 (18.2%) |
| Chronic Heart Failure – n(%) | 24 (18.0%) | 25 (18.2%) | 3 (20.0%) | 5 (33.3%) | 1 (9.1%) | 1 (9.1%) |
| **Baseline Lab Values** | | | | | | |
| WBC (10^9^/L) – Med [IQR] | 17.4  [9.5, 24.5] | 16.2 [10.5, 27.0] | 23.0 [12.0, 29.1] | 18.5 [14.1, 26.7] | 17.1 [12.8, 20.1] | 16.8 [9.3, 27.7] |
| Hemoglobin (g/dL) – Med [IQR] | 9.2 [8.4, 11.2] | 9.9 [8.8, 11.2] | 8.2 [7.4, 9.9] | 11.1  [8.6, 12.9] | 9.2 [8.4, 11.0] | 10.4 [9.3, 11.1] |
| K+ (mEq/L) – Med [IQR] | 4.2 [3.8, 4.7] | 4.2 [3.9, 4.8] | 4.4 [4.2, 4.7] | 4.0 [3.3, 4.8] | 4.4 [4.1, 4.9] | 4.6 [3.8, 4.9] |
| Creatinine (mg/dL) – Med [IQR] | 2.0 [1.3, 2.9] | 1.9 [1.2, 2.7] | 2.7 [1.9, 3.4] | 2.3 [1.2, 4.1] | 2.4 [1.3, 3.9] | 3.2 [2.2, 4.3] |
| BUN (mg/dL) – Med [IQR] | 26 [16 -44] | 24 [15, 41] | 36 [15, 52] | 25 [10, 38] | 22 [15, 35] | 22 [18, 30] |
| HCO3^-^ (mEq/L) – Med [IQR] | 20 [16, 22] | 19 [15, 23] | 18 [15, 21] | 20 [16, 22] | 19 [18, 23] | 16 [14, 18] |
| P/F Ratio (mmHg)– Med [IQR] | 208 [130, 291] | 211 [148, 280] | 218 [148, 314] | 310 [229, 367] | 228 [114, 280] | 196 [99, 326] |
| **Treatment Characteristics** | | | | | | |
| Duration of Study Drug Exposure (hr) – Med [IQR] | 48.0 [36.8, 49.2] | 48.1 [35.0, 49.3] | 48.0 [43.2, 49.2] | 47.3 [23.6, 48.4] | 46.8 [29.1, 49.1] | 40.6 [27.3, 48.0] |
| Fluid Administration (mL) – Med [IQR] | 607 [378. 919] | 429 [250, 629] | 543 [400, 808] | 455 [225, 936] | 538 [498, 664] | 536 [318, 687] |
| Baseline characteristics of the cohort, stratified by ACEi/ARB exposure and treatment assignment. Data reported as mean (SD) unless otherwise indicated. Abbreviations: ARB – angiotensin receptor blocker; ACEi – angiotensin-converting enzyme inhibitor; APACHE – acute physiology and chronic health evaluation score; ARDS – acute respiratory distress syndrome; RRT – renal replacement therapy; NED – norepinephrine equivalent dose; ScvO2 – central venous oxygen saturation; WBC – white blood cell count; K^+^ – potassium; HCO3^-^ – Bicarbonate; BUN – blood urea nitrogen; P/F Ratio –arterial oxygen tension to fraction inspired oxygen ratio. | | | | | | |

| **Table-S3: Prevalence of Missing Data** | | | | |
| --- | --- | --- | --- | --- |
| Variable | No ACEi/ARB | ACEi | ARB | Total |
| Exposure Status | 0 | 0 | 0 | 0 |
| Treatment Assignment | 0 | 0 | 0 | 0 |
| **Demographics and Clinical Factors** |  |  |  |  |
| Age | 0 | 0 | 0 | 0 |
| Female | 0 | 0 | 0 | 0 |
| Body Mass Index | 0 | 0 | 0 | 0 |
| Ideal Body Weight | 0 | 0 | 0 | 0 |
| Cause of Vasodilatory Shock | 0 | 0 | 0 | 0 |
| Baseline APACHE II Score | 0 | 0 | 0 | 0 |
| Baseline Albumin | 9 (3.3%) | 1 (3.3%) | 1 (4.5%) | 11 (3.4%) |
| ARDS at Baseline | 0 | 0 | 0 | 0 |
| Intubated at Baseline | 0 | 0 | 0 | 0 |
| RRT at Screening | 0 | 0 | 0 | 0 |
|  |  |  |  |  |
| **Baseline Cardiovascular Status** |  |  |  |  |
| Mean Arterial Pressure | 0 | 0 | 0 | 0 |
| Average NED in past 6 hours | 1 (0.4%) | 0 | 0 | 1 (0.3%) |
| Vasopressin use in past 6 hours | 0 | 0 | 0 | 0 |
| Central Venous Pressure | 59 (21.9%) | 6 (20.0%) | 7 (31.8%) | 72 (22.4%) |
| Cardiac Index | 154 (57.0%) | 15 (50.0%) | 10 (45.5%) | 179 (55.8%) |
| ScvO_2_ | 71 (26.3%) | 8 (26.7%) | 5 (22.7%) | 84 (26.2%) |
|  |  |  |  |  |
| **Medical History** |  |  |  |  |
| Hypertension | 0 | 0 | 0 | 0 |
| Chronic Kidney Disease | 0 | 0 | 0 | 0 |
| Diabetes | 0 | 0 | 0 | 0 |
| Coronary Artery Disease | 0 | 0 | 0 | 0 |
| Chronic Heart Failure | 0 | 0 | 0 | 0 |
|  |  |  |  |  |
| **Baseline Lab Values** |  |  |  |  |
| White Blood Cell Count | 1 (0.4%) | 0 | 0 | 1 (0.4%) |
| Hemoglobin | 1 (0.4%) | 0 | 0 | 1 (0.4%) |
| Potassium | 1 (0.4%) | 0 | 0 | 1 (0.4%) |
| Creatinine | 1 (0.4%) | 0 | 0 | 1 (0.4%) |
| Blood Urea Nitrogen | 2 (0.7%) | 0 | 0 | 2 (0.6%) |
| Bicarbonate | 14 (5.2%) | 0 | 0 | 14 (4.4%) |
| PaO2/FiO2 Ratio | 0 | 0 | 0 | 0 |
|  |  |  |  |  |
| **Treatment Characteristics** |  |  |  |  |
| Duration of Study Drug Exposure | 0 | 0 | 0 | 0 |
| Fluid Administration | 0 | 0 | 0 | 0 |
|  |  |  |  |  |
| **Biomarkers** |  |  |  |  |
| Renin Hour 0 | 39 (14.4%) | 3 (10.0%) | 1 (4.5%) | 43 (13.4%) |
| Renin Hour 3 | 92 (34.1%) | 9 (30.0%) | 4 (18.2%) | 104 (32.4%) |
| Angiotensin I Hour 0 | 31 (11.5%) | 2 (6.7%) | 2 (9.1%) | 35 (10.9%) |
| Angiotensin I Hour 3 | 25 (9.3%) | 2 (6.7%) | 2 (9.1%) | 28 (8.7%) |
| Angiotensin II Hour 0 | 33 (12.2%) | 2 (6.7%) | 2 (9.1%) | 37 (11.5%) |
| Angiotensin II Hour 3 | 25 (9.3%) | 2 (6.7%) | 2 (9.1%) | 28 (8.7%) |
| Prevalence of missing data overall and by ACEi/ARB exposure. All variables reported as n (%).  Abbreviations: ARB – angiotensin receptor blocker; ACEi – angiotensin-converting enzyme inhibitor; APACHE – acute physiology and chronic health evaluation score; ARDS – acute respiratory distress syndrome; RRT – renal replacement therapy; NED – norepinephrine equivalent dose; ScvO2 – central venous oxygen saturation | | | | |

| **Table-S4: Multivariable Models for MAP at Hour-1 by ACEi/ARB Group and Treatment** | | | | | | | | |
| --- | --- | --- | --- | --- | --- | --- | --- | --- |
|  | **Model 1** | | | | **Model 2** | | | |
| Variable | Estimate | 95%CI | | p-value | Estimate | 95%CI | | p-value |
| **ACEi vs. No ACEi/ARB** | **3.5** | **0.0** | **6.9** | **0.0494** | **3.3** | **-0.2** | **6.8** | **0.0684** |
| **ARB vs. No ACEi/ARB** | **-0.3** | **-4.2** | **3.6** | **0.88** | **-0.4** | **-4.3** | **3.6** | **0.86** |
| **Ang-II Treatment (vs. Placebo)** | **9.1** | **7.6** | **10.6** | **<0.0001** | **9.1** | **7.5** | **10.7** | **<0.0001** |
| **ACEi*Treatment Interaction** | **-2.2** | **-7.0** | **2.6** | **0.38** | **-2.4** | **-7.3** | **2.6** | **0.35** |
| **ARB*Treatment Interaction** | **-6.0** | **-11.5** | **-0.6** | **0.0299** | **-6.0** | **-11.6** | **-0.4** | **0.0362** |
| Baseline MAP (mmHg) | 0.6 | 0.4 | 0.7 | <0.0001 | 0.5 | 0.4 | 0.7 | <0.0001 |
| Age _(per 10 years)_ | 0.0 | -0.5 | 0.4 | 0.95 | -0.1 | -0.6 | 0.5 | 0.84 |
| Baseline NED _log(mcg/kg/min)_ | -2.5 | -4.3 | -0.7 | 0.0063 | -1.7 | -2.9 | -0.5 | 0.0065 |
| Female Sex | 1.2 | -0.3 | 2.6 | 0.11 | 1.0 | -0.5 | 2.5 | 0.17 |
| Baseline APACHE-II Score | -0.1 | -0.2 | 0.0 | 0.0730 | -0.1 | -0.2 | 0.0 | 0.15 |
| Baseline Albumin (g/dL) |  |  |  |  | 0.9 | -0.3 | 2.1 | 0.14 |
| ARDS at Baseline |  |  |  |  | -0.2 | -1.9 | 1.5 | 0.81 |
| Chronic Kidney Disease |  |  |  |  | -0.2 | -1.9 | 1.5 | 0.85 |
| Chronic Hypertension |  |  |  |  | 0.4 | -1.2 | 2.0 | 0.62 |
| RRT at Baseline |  |  |  |  | -0.5 | -2.1 | 1.2 | 0.58 |
| Multivariable model outputs for MAP at hour 1. Estimates indicate the difference in mmHg per unit change in the predictor variable. Abbreviations: ACEi – angiotensin-converting enzyme inhibitor; ARB – angiotensin receptor blocker; NED – norepinephrine equivalent dose; APACHE – acute physiology and chronic health evaluation score; ARDS – acute respiratory distress syndrome; RRT – renal replacement therapy; 95%CI – 95% confidence interval. | | | | | | | | |

| **Table-S5: Hour 0-3 Multivariable Longitudinal Models for Effect of Treatment on NED by ACEi/ARB Exposure** | | | | | | | | | |
| --- | --- | --- | --- | --- | --- | --- | --- | --- | --- |
|  | | **Model 1** | | | | **Model 2** | | | |
| Variable | Estimate | | 95%CI | | p-value | Estimate | 95%CI | | p-value |
| Intercept | 1.508 | | 0.566 | 2.451 |  | 2.014 | 0.625 | 3.402 |  |
| **Angiotensin-II Treatment** | **-0.020** | | **-0.180** | **0.139** | **0.80** | **-0.343** | **-0.571** | **-0.115** | **0.0031** |
| **Hour** | **0.011** | | **-0.003** | **0.024** | **0.13** | **-0.025** | **-0.026** | **-0.023** | **<0.0001** |
| **ACEi (vs. No ACEi/ARB)** | **0.101** | | **-0.264** | **0.466** | **0.59** | **0.116** | **-0.396** | **0.628** | **0.66** |
| **ARB (vs. No ACEi/ARB)** | **-0.138** | | **-0.548** | **0.272** | **0.51** | **-0.245** | **-0.814** | **0.324** | **0.40** |
| **Hour*Treatment Interaction** | **-0.067** | | **-0.086** | **-0.048** | **<0.0001** | **-0.002** | **-0.004** | **0.000** | **0.0884** |
| **ACEi*Treatment Interaction** | **-0.096** | | **-0.605** | **0.413** | **0.72** | **0.145** | **-0.568** | **0.859** | **0.69** |
| **ARB*Treatment Interaction** | **0.146** | | **-0.429** | **0.722** | **0.62** | **0.440** | **-0.373** | **1.253** | **0.29** |
| **Hour*ACEi Interaction** | **-0.016** | | **-0.059** | **0.028** | **0.48** | **-0.015** | **-0.020** | **-0.010** | **<0.0001** |
| **Hour*ARB Interaction** | **0.008** | | **-0.041** | **0.056** | **0.76** | **0.008** | **0.004** | **0.012** | **0.0001** |
| **Hour*ACEi*Treatment Interaction** | **-0.038** | | **-0.100** | **0.024** | **0.23** | **-0.009** | **-0.016** | **-0.002** | **0.0138** |
| **Hour*ARB*Treatment Interaction** | **0.024** | | **-0.045** | **0.093** | **0.49** | **-0.017** | **-0.024** | **-0.010** | **<0.0001** |
| Age (years) | -0.004 | | -0.009 | 0.001 | 0.0980 | 0.000 | -0.007 | 0.007 | 0.96 |
| Female Sex | 0.112 | | -0.036 | 0.260 | 0.14 | 0.100 | -0.108 | 0.308 | 0.35 |
| Baseline MAP (mmHg) | -0.040 | | -0.053 | -0.026 | <0.0001 | -0.048 | -0.067 | -0.029 | <0.0001 |
| Baseline APACHE-II Score | 0.011 | | 0.002 | 0.020 | 0.0116 | 0.019 | 0.006 | 0.032 | 0.0047 |
| Baseline Albumin (g/dL) |  | |  |  |  | -0.094 | -0.167 | -0.021 | 0.0117 |
| ARDS at Baseline |  | |  |  |  | 0.009 | -0.099 | 0.117 | 0.87 |
| Chronic Kidney Disease |  | |  |  |  | 0.014 | -0.092 | 0.121 | 0.79 |
| Chronic Hypertension |  | |  |  |  | 0.063 | -0.039 | 0.165 | 0.23 |
| RRT at Baseline |  | |  |  |  | 0.108 | 0.005 | 0.211 | 0.0393 |
| Multivariable mixed effects model output for NED over time *during* the active titration period. Estimates indicate the difference in log(mcg/kg/min) per unit change in the predictor variable. Abbreviations: ACEi – angiotensin-converting enzyme inhibitor; ARB – angiotensin receptor blocker; NED – norepinephrine equivalent dose; APACHE – acute physiology and chronic health evaluation score; ARDS – acute respiratory distress syndrome; RRT – renal replacement therapy; 95%CI – 95% confidence interval. | | | | | | | | | |

| **Table-S6: Hour 4-48 Multivariable Longitudinal Models for Effect of Treatment on NED by ACEi/ARB Exposure** | | | | | | | | | |
| --- | --- | --- | --- | --- | --- | --- | --- | --- | --- |
|  | | **Model 1** | | | | **Model 2** | | | |
| Variable | Estimate | | 95%CI | | p-value | Estimate | 95%CI | | p-value |
| Intercept | 1.938 | | 0.618 | 3.257 |  | 2.014 | 0.625 | 3.402 |  |
| **Angiotensin-II Treatment** | **-0.328** | | **-0.549** | **-0.106** | **0.0037** | **-0.343** | **-0.571** | **-0.115** | **0.0031** |
| **Hour** | **-0.025** | | **-0.026** | **-0.024** | **<0.0001** | **-0.025** | **-0.026** | **-0.023** | **<0.0001** |
| **ACEi (vs. No ACEi/ARB)** | **0.093** | | **-0.412** | **0.597** | **0.72** | **0.116** | **-0.396** | **0.628** | **0.66** |
| **ARB (vs. No ACEi/ARB)** | **-0.184** | | **-0.748** | **0.381** | **0.52** | **-0.245** | **-0.814** | **0.324** | **0.40** |
| **Hour*Treatment Interaction** | **0.000** | | **-0.002** | **0.002** | **0.99** | **-0.002** | **-0.004** | **0.000** | **0.0884** |
| **ACEi*Treatment Interaction** | **0.092** | | **-0.616** | **0.801** | **0.80** | **0.145** | **-0.568** | **0.859** | **0.69** |
| **ARB*Treatment Interaction** | **0.667** | | **-0.128** | **1.463** | **0.10** | **0.440** | **-0.373** | **1.253** | **0.29** |
| **Hour*ACEi Interaction** | **-0.015** | | **-0.019** | **-0.010** | **<0.0001** | **-0.015** | **-0.020** | **-0.010** | **<0.0001** |
| **Hour*ARB Interaction** | **0.008** | | **0.004** | **0.012** | **<0.0001** | **0.008** | **0.004** | **0.012** | **0.0001** |
| **Hour*ACEi*Treatment Interaction** | **-0.010** | | **-0.017** | **-0.003** | **0.0031** | **-0.009** | **-0.016** | **-0.002** | **0.0138** |
| **Hour*ARB*Treatment Interaction** | **-0.019** | | **-0.026** | **-0.012** | **<0.0001** | **-0.017** | **-0.024** | **-0.010** | **<0.0001** |
| Age (years) | -0.004 | | -0.010 | 0.003 | 0.25 | 0.000 | -0.007 | 0.007 | 0.96 |
| Female Sex | 0.108 | | -0.098 | 0.314 | 0.31 | 0.100 | -0.108 | 0.308 | 0.35 |
| Baseline MAP (mmHg) | -0.053 | | -0.072 | -0.034 | <0.0001 | -0.048 | -0.067 | -0.029 | <0.0001 |
| Baseline APACHE-II Score | 0.027 | | 0.015 | 0.039 | <0.0001 | 0.019 | 0.006 | 0.032 | 0.0047 |
| Baseline Albumin (g/dL) |  | |  |  |  | -0.238 | -0.405 | -0.072 | 0.005 |
| ARDS at Baseline |  | |  |  |  | 0.140 | -0.106 | 0.386 | 0.26 |
| Chronic Hypertension |  | |  |  |  | 0.059 | -0.175 | 0.293 | 0.62 |
| Chronic Kidney Disease |  | |  |  |  | -0.142 | -0.386 | 0.101 | 0.25 |
| RRT at Baseline |  | |  |  |  | 0.254 | 0.020 | 0.487 | 0.0336 |
| Multivariable mixed effects model output for NED over time *after* the active titration period. Estimates indicate the difference in log(mcg/kg/min) per unit change in the predictor variable. Abbreviations: ACEi – angiotensin-converting enzyme inhibitor; ARB – angiotensin receptor blocker; NED – norepinephrine equivalent dose; APACHE – acute physiology and chronic health evaluation score; ARDS – acute respiratory distress syndrome; RRT – renal replacement therapy; 95%CI – 95% confidence interval. | | | | | | | | | |

| **Table-S7: Hour 0-3 Multivariable Longitudinal Models for Effect of Treatment on Study Drug Dose by ACEi/ARB Exposure** | | | | | | | | | |
| --- | --- | --- | --- | --- | --- | --- | --- | --- | --- |
|  | | **Model 1** | | | | **Model 2** | | | |
| Variable | Estimate | | 95%CI | | p-value | Estimate | 95%CI | | p-value |
| Intercept | 23.7 | | 4.6 | 42.8 |  | 224.40 | 182.02 | 266.77 |  |
| **Angiotensin-II Treatment** | **-14.6** | | **-23.5** | **-5.7** | **0.0013** | **-12.52** | **-20.88** | **-4.15** | **0.0034** |
| **Hour** | **33.6** | | **31.3** | **35.9** | **<0.0001** | **33.67** | **31.37** | **35.97** | **<0.0001** |
| **ACEi (vs. No ACEi/ARB)** | **-4.1** | | **-24.5** | **16.3** | **0.69** | **-0.86** | **-19.75** | **18.03** | **0.93** |
| **ARB (vs. No ACEi/ARB)** | **-4.1** | | **-27.1** | **19.0** | **0.73** | **-4.60** | **-25.92** | **16.72** | **0.67** |
| **Hour*Treatment Interaction** | **-29.7** | | **-32.9** | **-26.5** | **<0.0001** | **-30.26** | **-33.51** | **-27.00** | **<0.0001** |
| **ACEi*Treatment Interaction** | **3.7** | | **-24.7** | **32.1** | **0.80** | **6.82** | **-19.58** | **33.22** | **0.61** |
| **ARB*Treatment Interaction** | **11.6** | | **-20.7** | **43.9** | **0.48** | **9.14** | **-21.20** | **39.48** | **0.55** |
| **Hour*ACEi Interaction** | **-7.9** | | **-15.3** | **-0.5** | **0.0364** | **-8.00** | **-15.40** | **-0.61** | **0.0339** |
| **Hour*ARB Interaction** | **0.1** | | **-8.7** | **9.0** | **0.97** | **0.04** | **-8.77** | **8.86** | **0.99** |
| **Hour*ACEi*Treatment Interaction** | **9.0** | | **-1.3** | **19.3** | **0.0856** | **10.30** | **-0.15** | **20.75** | **0.0533** |
| **Hour*ARB*Treatment Interaction** | **24.0** | | **11.9** | **36.1** | **0.0001** | **21.37** | **9.04** | **33.69** | **0.0007** |
| Age (years) | 0.0 | | -0.3 | 0.2 | 0.81 | 0.02 | -0.21 | 0.24 | 0.89 |
| Female Sex | -4.9 | | -12.0 | 2.2 | 0.18 | -1.52 | -7.85 | 4.81 | 0.64 |
| Baseline NED _log(mcg/kg/min)_ | 20.8 | | 12.1 | 29.5 | <0.0001 | 5.20 | -0.01 | 10.41 | 0.0506 |
| Baseline APACHE-II Score | 0.1 | | -0.3 | 0.5 | 0.70 | 0.02 | -0.38 | 0.41 | 0.94 |
| Baseline MAP (mmHg) |  | |  |  |  | -2.76 | -3.36 | -2.16 | <0.0001 |
| Baseline Albumin (g/dL) |  | |  |  |  | -3.70 | -8.82 | 1.41 | 0.16 |
| ARDS at Baseline |  | |  |  |  | -0.01 | -7.50 | 7.48 | 0.99 |
| Chronic Hypertension |  | |  |  |  | -1.13 | -8.19 | 5.93 | 0.75 |
| Chronic Kidney Disease |  | |  |  |  | 3.80 | -3.57 | 11.16 | 0.31 |
| RRT at Baseline |  | |  |  |  | 1.84 | -5.28 | 8.97 | 0.61 |
| Multivariable mixed effects model outputs for study drug dose over time *during* the active titration period. Estimates indicate the difference in ng/kg/min per unit change in the predictor variable. Abbreviations: ACEi – angiotensin-converting enzyme inhibitor; ARB – angiotensin receptor blocker; NED – norepinephrine equivalent dose; APACHE – acute physiology and chronic health evaluation score; MAP – mean arterial pressure; ARDS – acute respiratory distress syndrome; RRT – renal replacement therapy; 95%CI – 95% confidence interval. | | | | | | | | | |

| **Table-S8: Hour 4-48 Multivariable Longitudinal Model for Effect of Treatment on Study Drug Dose by ACEi/ARB Exposure** | | | | | | | | | |
| --- | --- | --- | --- | --- | --- | --- | --- | --- | --- |
|  | | **Model 1** | | | | **Model 2** | | | |
| Variable | Estimate | | 95%CI | | p-value | Estimate | 95%CI | | p-value |
| Intercept | 33.2 | | 23.3 | 43.2 |  | 56.53 | 30.74 | 82.32 |  |
| **Angiotensin-II Treatment** | **-17.8** | | **-21.8** | **-13.8** | **<0.0001** | **-17.49** | **-21.63** | **-13.36** | **<0.0001** |
| **Hour** | **-0.2** | | **-0.2** | **-0.2** | **<0.0001** | **-0.17** | **-0.19** | **-0.15** | **<0.0001** |
| **ACEi (vs. No ACEi/ARB)** | **-8.0** | | **-17.1** | **1.0** | **0.0822** | **-4.46** | **-13.70** | **4.78** | **0.34** |
| **ARB (vs. No ACEi/ARB)** | **2.0** | | **-9.2** | **13.1** | **0.73** | **2.43** | **-8.81** | **13.66** | **0.67** |
| **Hour*Treatment Interaction** | **0.1** | | **0.1** | **0.2** | **<0.0001** | **0.14** | **0.11** | **0.16** | **<0.0001** |
| **ACEi*Treatment Interaction** | **6.0** | | **-6.7** | **18.7** | **0.36** | **6.03** | **-6.83** | **18.90** | **0.36** |
| **ARB*Treatment Interaction** | **11.1** | | **-3.9** | **26.1** | **0.15** | **9.81** | **-5.53** | **25.16** | **0.21** |
| **Hour*ACEi Interaction** | **0.1** | | **0.0** | **0.2** | **0.0002** | **0.12** | **0.07** | **0.17** | **<0.0001** |
| **Hour*ARB Interaction** | **-0.1** | | **-0.1** | **0.0** | **0.0044** | **-0.09** | **-0.15** | **-0.04** | **0.0016** |
| **Hour*ACEi*Treatment Interaction** | **-0.2** | | **-0.3** | **-0.1** | **<0.0001** | **-0.28** | **-0.35** | **-0.20** | **<0.0001** |
| **Hour*ARB*Treatment Interaction** | **0.2** | | **0.1** | **0.2** | **0.0008** | **0.15** | **0.06** | **0.24** | **0.0008** |
| Age (years) | -0.1 | | -0.2 | 0.0 | 0.11 | -0.09 | -0.22 | 0.05 | 0.20 |
| Female Sex | 0.4 | | -3.4 | 4.2 | 0.84 | 1.15 | -2.68 | 4.98 | 0.56 |
| Baseline NED _log(mcg/kg/min)_ | 3.9 | | -0.9 | 8.6 | 0.11 | 1.87 | -1.28 | 5.02 | 0.24 |
| Baseline APACHE-II Score | 0.3 | | 0.1 | 0.5 | 0.0113 | 0.24 | 0.00 | 0.48 | 0.0516 |
| Baseline MAP (mmHg) |  | |  |  |  | -0.25 | -0.61 | 0.12 | 0.19 |
| Baseline Albumin (g/dL) |  | |  |  |  | -1.78 | -4.85 | 1.29 | 0.26 |
| ARDS at Baseline |  | |  |  |  | 0.28 | -4.24 | 4.80 | 0.90 |
| Chronic Hypertension |  | |  |  |  | 0.17 | -4.09 | 4.43 | 0.94 |
| Chronic Kidney Disease |  | |  |  |  | -0.68 | -5.14 | 3.77 | 0.76 |
| RRT at Baseline |  | |  |  |  | 0.67 | -3.66 | 4.99 | 0.76 |
| Multivariable mixed effects model outputs for study drug dose over time *after* the active titration period. Estimates indicate the difference in ng/kg/min per unit change in the predictor variable. Abbreviations: ACEi – angiotensin-converting enzyme inhibitor; ARB – angiotensin receptor blocker; NED – norepinephrine equivalent dose; APACHE – acute physiology and chronic health evaluation score; MAP – mean arterial pressure; ARDS – acute respiratory distress syndrome; RRT – renal replacement therapy; 95%CI – 95% confidence interval. | | | | | | | | | |

| **Table-S9: Baseline Renin as Function of ACEi/ARB Group** | | | | | | | | |
| --- | --- | --- | --- | --- | --- | --- | --- | --- |
|  | **Model 1** | | | | **Model 2** | | | |
| Variable | Estimate | 95%CI | | p-value | Estimate | 95%CI | | p-value |
| **ACEi vs. No ACEi/ARB** | **0.95** | **0.36** | **1.54** | **0.0016** | **6.83** | **4.42** | **9.25** | **<0.0001** |
| **ARB vs. No ACEi/ARB** | **0.71** | **0.05** | **1.37** | **0.0341** | **0.99** | **0.38** | **1.60** | **0.0017** |
| Age _(per 10 years)_ | -0.17 | -0.28 | -0.05 | 0.0038 | 0.58 | -0.11 | 1.27 | 0.0964 |
| Female Sex | -0.34 | -0.69 | 0.02 | 0.0607 | -0.14 | -0.27 | -0.02 | 0.0275 |
| Baseline NED _log(mcg/kg/min)_ | 0.66 | 0.38 | 0.93 | <0.0001 | -0.34 | -0.69 | 0.02 | 0.0627 |
| Baseline APACHE-II Score | 0.03 | 0.01 | 0.05 | 0.0095 | 0.59 | 0.29 | 0.88 | 0.0001 |
| Baseline MAP _(mmHg)_ |  |  |  |  | 0.02 | -0.01 | 0.04 | 0.14 |
| Baseline Albumin _(g/dL)_ |  |  |  |  | -0.02 | -0.06 | 0.01 | 0.19 |
| ARDS at Baseline |  |  |  |  | 0.15 | -0.13 | 0.44 | 0.30 |
| Chronic Kidney Disease |  |  |  |  | 0.24 | -0.18 | 0.66 | 0.27 |
| Chronic Hypertension |  |  |  |  | -0.58 | -0.98 | -0.17 | 0.0059 |
| RRT at Baseline |  |  |  |  | 0.37 | -0.03 | 0.78 | 0.0671 |
| Multivariable model outputs for baseline renin. Estimates indicate the difference in Log(pg/mL) per unit change in the predictor variable. Abbreviations: ACEi – angiotensin-converting enzyme inhibitor; ARB – angiotensin receptor blocker; NED – norepinephrine equivalent dose; APACHE – acute physiology and chronic health evaluation score; ARDS – acute respiratory distress syndrome; RRT – renal replacement therapy; 95%CI – 95% confidence interval. | | | | | | | | |

| **Table-S10: Baseline Ang-I as Function of ACEi/ARB Group** | | | | | | | | |
| --- | --- | --- | --- | --- | --- | --- | --- | --- |
|  | **Model 1** | | | | **Model 2** | | | |
| Variable | Estimate | 95%CI | | p-value | Estimate | 95%CI | | p-value |
| **ACEi vs. No ACEi/ARB** | **1.15** | **0.57** | **1.74** | **0.0001** | **1.29** | **0.67** | **1.91** | **<0.0001** |
| **ARB vs. No ACEi/ARB** | **0.63** | **-0.05** | **1.31** | **0.0709** | **0.52** | **-0.18** | **1.23** | **0.1445** |
| Age _(per 10 years)_ | -0.05 | -0.16 | 0.07 | 0.41 | -0.05 | -0.18 | 0.07 | 0.40 |
| Female Sex | -0.07 | -0.42 | 0.28 | 0.70 | -0.13 | -0.49 | 0.22 | 0.47 |
| Baseline NED _log(mcg/kg/min)_ | 0.31 | 0.03 | 0.59 | 0.0300 | 0.27 | -0.02 | 0.57 | 0.0719 |
| Baseline APACHE-II Score | 0.00 | -0.02 | 0.02 | 0.71 | 0.01 | -0.02 | 0.03 | 0.61 |
| Baseline MAP _(mmHg)_ |  |  |  |  | 0.00 | -0.04 | 0.03 | 0.77 |
| Baseline Albumin _(g/dL)_ |  |  |  |  | -0.07 | -0.36 | 0.22 | 0.62 |
| ARDS at Baseline |  |  |  |  | 0.22 | -0.21 | 0.64 | 0.32 |
| Chronic Kidney Disease |  |  |  |  | -0.70 | -1.12 | -0.28 | 0.0011 |
| Chronic Hypertension |  |  |  |  | 0.38 | -0.03 | 0.78 | 0.0662 |
| RRT at Baseline |  |  |  |  | 0.00 | -0.40 | 0.40 | 0.99 |
| Multivariable model outputs for baseline Ang-I. Estimates indicate the difference in Log(pg/mL) per unit change in the predictor variable. Abbreviations: Ang-I – angiotensin-I; ACEi – angiotensin-converting enzyme inhibitor; ARB – angiotensin receptor blocker; NED – norepinephrine equivalent dose; APACHE – acute physiology and chronic health evaluation score; ARDS – acute respiratory distress syndrome; RRT – renal replacement therapy; 95%CI – 95% confidence interval. | | | | | | | | |

| **Table-S11: Baseline Ang-II as Function of ACEi/ARB Group** | | | | | | | | |
| --- | --- | --- | --- | --- | --- | --- | --- | --- |
|  | **Model 1** | | | | **Model 2** | | | |
| Variable | Estimate | 95%CI | | p-value | Estimate | 95%CI | | p-value |
| **ACEi vs. No ACEi/ARB** | **-0.86** | **-1.47** | **-0.25** | **0.0058** | **-0.61** | **-1.25** | **0.04** | **0.0651** |
| **ARB vs. No ACEi/ARB** | **0.75** | **0.04** | **1.46** | **0.0377** | **0.79** | **0.06** | **1.53** | **0.0348** |
| Age _(per 10 years)_ | -0.04 | -0.16 | 0.08 | 0.49 | 0.04 | -0.09 | 0.18 | 0.5282 |
| Female Sex | -0.14 | -0.51 | 0.23 | 0.46 | -0.16 | -0.53 | 0.22 | 0.4033 |
| Baseline NED _log(mcg/kg/min)_ | 0.11 | -0.18 | 0.40 | 0.47 | 0.08 | -0.24 | 0.39 | 0.6344 |
| Baseline APACHE-II Score | -0.02 | -0.05 | 0.00 | 0.0395 | -0.02 | -0.05 | 0.00 | 0.0477 |
| Baseline MAP _(mmHg)_ |  |  |  |  | 0.00 | -0.03 | 0.04 | 0.7818 |
| Baseline Albumin _(g/dL)_ |  |  |  |  | -0.27 | -0.58 | 0.03 | 0.0758 |
| ARDS at Baseline |  |  |  |  | 0.43 | -0.02 | 0.88 | 0.062 |
| Chronic Kidney Disease |  |  |  |  | -0.35 | -0.79 | 0.09 | 0.1183 |
| Chronic Hypertension |  |  |  |  | -0.27 | -0.70 | 0.15 | 0.2097 |
| RRT at Baseline |  |  |  |  | 0.00 | -0.42 | 0.42 | 0.9992 |
| Multivariable model outputs for baseline Ang-II. Estimates indicate the difference in Log(pg/mL) per unit change in the predictor variable. Abbreviations: Ang-II – angiotensin-II; ACEi – angiotensin-converting enzyme inhibitor; ARB – angiotensin receptor blocker; NED – norepinephrine equivalent dose; APACHE – acute physiology and chronic health evaluation score; ARDS – acute respiratory distress syndrome; RRT – renal replacement therapy. | | | | | | | | |

| **Table-S12: Baseline Ang-I/Ang-II Ratio as Function of ACEi/ARB Group** | | | | | | | | |
| --- | --- | --- | --- | --- | --- | --- | --- | --- |
|  | **Model 1** | | | | **Model 2** | | | |
| Variable | Estimate | 95%CI | | p-value | Estimate | 95%CI | | p-value |
| **ACEi vs. No ACEi/ARB** | **2.01** | **1.50** | **2.53** | **<0.0001** | **1.90** | **1.38** | **2.42** | **<0.0001** |
| **ARB vs. No ACEi/ARB** | **-0.12** | **-0.72** | **0.48** | **0.69** | **-0.27** | **-0.87** | **0.32** | **0.37** |
| Age _(per 10 years)_ | -0.01 | -0.11 | 0.09 | 0.87 | -0.10 | -0.20 | 0.01 | 0.0800 |
| Female Sex | 0.05 | -0.26 | 0.37 | 0.74 | 0.01 | -0.29 | 0.32 | 0.94 |
| Baseline NED _log(mcg/kg/min)_ | 0.19 | -0.05 | 0.44 | 0.12 | 0.19 | -0.06 | 0.44 | 0.14 |
| Baseline APACHE-II Score | 0.03 | 0.01 | 0.04 | 0.0066 | 0.03 | 0.01 | 0.05 | 0.0042 |
| Baseline MAP _(mmHg)_ |  |  |  |  | -0.01 | -0.04 | 0.01 | 0.34 |
| Baseline Albumin _(g/dL)_ |  |  |  |  | 0.20 | -0.04 | 0.45 | 0.1084 |
| ARDS at Baseline |  |  |  |  | -0.25 | -0.61 | 0.11 | 0.18 |
| Chronic Kidney Disease |  |  |  |  | -0.39 | -0.75 | -0.04 | 0.0293 |
| Chronic Hypertension |  |  |  |  | 0.68 | 0.33 | 1.02 | 0.0001 |
| RRT at Baseline |  |  |  |  | 0.00 | -0.34 | 0.34 | 0.99 |
| Multivariable model output for baseline Ang-I/Ang-II Ratio. Estimates indicate the difference in Log(arbitrary units) per unit change in the predictor variable. Abbreviations: Ang – angiotensin; ACEi – angiotensin-converting enzyme inhibitor; ARB – angiotensin receptor blocker; NED – norepinephrine equivalent dose; APACHE – acute physiology and chronic health evaluation score; ARDS – acute respiratory distress syndrome; RRT – renal replacement therapy; 95%CI – 95% confidence interval. | | | | | | | | |

| **Table-S13: Change in Log-Renin as Function of ACEi/ARB Group and Ang-II Treatment** | | | | | | | | |
| --- | --- | --- | --- | --- | --- | --- | --- | --- |
|  | **Model 1** | | | | **Model 2** | | | |
| Variable | Estimate | 95%CI | | p-value | Estimate | 95%CI | | p-value |
| **Ang-II Treatment _(No ACEi/ARB)_** | **-0.59** | **-0.73** | **-0.44** | **<0.0001** | **-0.55** | **-0.71** | **-0.40** | **<0.0001** |
| **ACEi vs. No ACEi/ARB _(Placebo)_** | **-0.05** | **-0.37** | **0.26** | **0.75** | **-0.04** | **-0.37** | **0.29** | **0.80** |
| **ARB vs. No ACEi/ARB _(Placebo)_** | **-0.12** | **-0.44** | **0.20** | **0.46** | **-0.10** | **-0.43** | **0.24** | **0.57** |
| **ACEi*Treatment Interaction** | **-0.14** | **-0.58** | **0.30** | **0.52** | **-0.21** | **-0.67** | **0.25** | **0.38** |
| **ARB*Treatment Interaction** | **0.58** | **0.10** | **1.07** | **0.0196** | **0.69** | **0.16** | **1.21** | **0.0108** |
| Age _(per 10 years)_ | -0.02 | -0.06 | 0.02 | 0.87 | -0.02 | -0.07 | 0.03 | 0.045 |
| Baseline NED _log(mcg/kg/min)_ | 0.10 | -0.01 | 0.21 | 0.0648 | 0.12 | 0.00 | 0.24 | 0.0565 |
| Baseline APACHE-II Score | 0.00 | -0.01 | 0.01 | 0.70 | 0.00 | -0.01 | 0.01 | 0.69 |
| Baseline MAP _(mmHg)_ |  |  |  |  | 0.00 | -0.01 | 0.02 | 0.68 |
| Baseline Albumin _(g/dL)_ |  |  |  |  | -0.03 | -0.15 | 0.09 | 0.64 |
| ARDS at Baseline |  |  |  |  | -0.06 | -0.22 | 0.11 | 0.50 |
| Chronic Kidney Disease |  |  |  |  | 0.14 | -0.02 | 0.30 | 0.0948 |
| Chronic Hypertension |  |  |  |  | -0.06 | -0.22 | 0.09 | 0.41 |
| RRT at Baseline |  |  |  |  | 0.02 | -0.14 | 0.17 | 0.83 |
| Multivariable model output for the change in renin at hour 3. Estimates indicate the difference in Log(pg/mL) per unit change in the predictor variable. Estimates for interaction effects indicate the change in the estimate for the indicated drug exposure when treated with Ang-II instead of placebo. Abbreviations: Ang-II – angiotensin-II; ACEi – angiotensin-converting enzyme inhibitor; ARB – angiotensin receptor blocker; NED – norepinephrine equivalent dose; APACHE – acute physiology and chronic health evaluation score; ARDS – acute respiratory distress syndrome; RRT – renal replacement therapy; 95%CI – 95% confidence interval. | | | | | | | | |

| **Table-s14: Hour-1 MAP as a Function of Losartan Dose Equivalents** | | | | | | | | |
| --- | --- | --- | --- | --- | --- | --- | --- | --- |
|  | **Model 1** | | | | **Model 2** | | | |
| Variable | Estimate | 95%CI | | p-value | Estimate | 95%CI | | p-value |
| **ARB Dose (Placebo) _log(mg)_** | **-0.1** | **-1.0** | **0.7** | **0.76** | **-0.1** | **-1.0** | **0.8** | **0.81** |
| **Ang-II Treatment (vs. Placebo)** | **9.1** | **7.6** | **10.6** | **<0.0001** | **9.1** | **7.5** | **10.6** | **<0.0001** |
| **ARB Dose*Treatment Interaction** | **-1.2** | **-2.4** | **0.0** | **0.0585** | **-1.2** | **-2.4** | **0.1** | **0.0717** |
| Baseline MAP _(mmHg)_ | 0.6 | 0.4 | 0.7 | <0.0001 | 0.5 | 0.4 | 0.7 | <0.0001 |
| Age _(per 10 years)_ | 0.1 | -0.4 | 0.6 | 0.74 | 0.0 | -0.5 | 0.6 | 0.96 |
| Female Sex | 1.1 | -0.3 | 2.6 | 0.13 | 1.0 | -0.5 | 2.5 | 0.21 |
| Baseline NED _log(mcg/kg/min)_ | -1.9 | -3.1 | -0.7 | 0.0016 | -1.9 | -3.1 | -0.6 | 0.0036 |
| Baseline APACHE-II Score | -0.1 | -0.2 | 0.0 | 0.0927 | -0.1 | -0.2 | 0.0 | 0.19 |
| Baseline Albumin _(g/dL)_ |  |  |  |  | 1.0 | -0.3 | 2.2 | 0.12 |
| ARDS at Baseline |  |  |  |  | -0.2 | -2.0 | 1.6 | 0.82 |
| Chronic Hypertension |  |  |  |  | -0.1 | -1.8 | 1.6 | 0.89 |
| Chronic Kidney Disease |  |  |  |  | 0.0 | -1.8 | 1.8 | 0.97 |
| RRT at Baseline |  |  |  |  | -0.5 | -2.3 | 1.2 | 0.55 |
| Multivariable model outputs for MAP at hour 1 where ARB exposure was modeled as continuous variable based on last recorded dose. Estimates indicate the difference in mmHg per unit change in the predictor variable. Estimates for interaction effects indicate the change in the estimate when treated with Ang-II instead of placebo. The models excluded the n=29 ACEi-exposed patients (total sample n=292). Losartan equivalents were modeled on a natural log scale to accommodate observed distributions. Unexposed patients were considered to have an ARB dose = 0 Log(mg). Abbreviations: ACEi – angiotensin-converting enzyme inhibitor; ARB – angiotensin receptor blocker; MAP – mean arterial pressure; NED – norepinephrine equivalent dose; APACHE – acute physiology and chronic health evaluation score; ARDS – acute respiratory distress syndrome; RRT – renal replacement therapy; 95%CI – 95% confidence interval. | | | | | | | | |

| **Table-s15: Hour-6 NED as a Function of Losartan Dose Equivalents** | | | | | | | | |
| --- | --- | --- | --- | --- | --- | --- | --- | --- |
|  | **Model 1** | | | | **Model 2** | | | |
| Variable | Estimate | 95%CI | | p-value | Estimate | 95%CI | | p-value |
| **ARB Dose (Placebo) _log(mg)_** | **-0.008** | **-0.091** | **0.076** | **0.86** | **-0.009** | **-0.095** | **0.078** | **0.84** |
| **Ang-II Treatment (vs. Placebo)** | **-0.416** | **-0.561** | **-0.270** | **<0.0001** | **-0.460** | **-0.612** | **-0.307** | **<0.0001** |
| **ARB Dose*Treatment Interaction** | **0.116** | **0.002** | **0.231** | **0.0457** | **0.137** | **0.018** | **0.257** | **0.0248** |
| Baseline MAP _(mmHg)_ | -0.010 | -0.023 | 0.004 | 0.17 | -0.008 | -0.023 | 0.006 | 0.25 |
| Age _(per 10 years)_ | 0.000 | -0.046 | 0.046 | 0.99 | 0.016 | -0.037 | 0.068 | 0.56 |
| Female Sex | -0.033 | -0.178 | 0.112 | 0.65 | -0.009 | -0.159 | 0.142 | 0.91 |
| Baseline NED _log(mcg/kg/min)_ | 1.085 | 0.964 | 1.206 | <0.0001 | 1.075 | 0.946 | 1.205 | <0.0001 |
| Baseline APACHE-II Score | 0.005 | -0.003 | 0.014 | 0.23 | 0.006 | -0.004 | 0.015 | 0.24 |
| Baseline Albumin _(g/dL)_ |  |  |  |  | -0.109 | -0.229 | 0.012 | 0.0772 |
| ARDS at Baseline |  |  |  |  | -0.093 | -0.265 | 0.080 | 0.29 |
| Chronic Hypertension |  |  |  |  | 0.065 | -0.101 | 0.231 | 0.44 |
| Chronic Kidney Disease |  |  |  |  | 0.006 | -0.169 | 0.182 | 0.94 |
| RRT at Baseline |  |  |  |  | -0.009 | -0.179 | 0.160 | 0.91 |
| Multivariable model outputs for NED at hour 1 where ARB exposure was modeled as continuous variable based on last recorded dose. Estimates indicate the difference in log(mcg/kg/min) per unit change in the predictor variable. Estimates for interaction effects indicate the change in the estimate when treated with Ang-II instead of placebo. The models excluded the n=29 ACEi-exposed patients (total sample n=292). Losartan equivalents were modeled on a natural log scale to accommodate observed distributions. Unexposed patients were considered to have an ARB dose = 0 Log(mg). Abbreviations: ACEi – angiotensin-converting enzyme inhibitor; ARB – angiotensin receptor blocker; MAP – mean arterial pressure; NED – norepinephrine equivalent dose; APACHE – acute physiology and chronic health evaluation score; ARDS – acute respiratory distress syndrome; RRT – renal replacement therapy; 95%CI – 95% confidence interval. | | | | | | | | |

| **Table-s16: Hour-3 Study Drug Dose as a Function of Losartan Dose Equivalents** | | | | | | | | |
| --- | --- | --- | --- | --- | --- | --- | --- | --- |
|  | **Model 1** | | | | **Model 2** | | | |
| Variable | Estimate | 95%CI | | p-value | Estimate | 95%CI | | p-value |
| **ARB Dose (Placebo) _log(mg)_** | **2.0** | **-5.2** | **9.2** | **0.59** | **1.3** | **-6.1** | **8.6** | **0.73** |
| **Ang-II Treatment (vs. Placebo)** | **-89.3** | **-100.9** | **-77.8** | **<0.0001** | **-92.1** | **-104.1** | **-80.0** | **<0.0001** |
| **ARB Dose*Treatment Interaction** | **13.7** | **4.1** | **23.3** | **0.0053** | **13.0** | **3.0** | **23.0** | **0.0108** |
| Baseline Study Drug Dose _(ng/kg/min)_ | 2.7 | 0.5 | 5.0 | 0.0167 | 2.8 | 0.6 | 5.1 | 0.0146 |
| Baseline MAP _(mmHg)_ | -3.0 | -4.0 | -1.9 | <0.0001 | -2.8 | -3.9 | -1.7 | <0.0001 |
| Age _(per 10 years)_ | -1.8 | -5.5 | 1.8 | 0.32 | -1.3 | -5.4 | 2.8 | 0.54 |
| Female Sex | -0.7 | -12.3 | 10.8 | 0.90 | 1.6 | -10.2 | 13.5 | 0.79 |
| Baseline NED _log(mcg/kg/min)_ | 14.4 | 5.1 | 23.7 | 0.0026 | 12.4 | 2.6 | 22.1 | 0.0132 |
| Baseline APACHE-II Score | -0.1 | -0.7 | 0.6 | 0.82 | -0.2 | -0.9 | 0.6 | 0.63 |
| Baseline Albumin _(g/dL)_ |  |  |  |  | -9.3 | -18.7 | 0.1 | 0.0536 |
| ARDS at Baseline |  |  |  |  | -3.1 | -16.6 | 10.4 | 0.65 |
| Chronic Hypertension |  |  |  |  | -1.3 | -14.3 | 11.7 | 0.85 |
| Chronic Kidney Disease |  |  |  |  | 2.9 | -11.0 | 16.8 | 0.68 |
| RRT at Baseline |  |  |  |  | 1.3 | -12.0 | 14.7 | 0.85 |
| Multivariable model outputs for Study Drug Dose at hour 3 (end of active titration period) where ARB exposure was modeled as continuous variable based on last recorded dose. Estimates indicate the difference in ng/kg/min per unit change in the predictor variable. Estimates for interaction effects indicate the change in the estimate when treated with Ang-II instead of placebo. The models excluded the n=29 ACEi-exposed patients (total sample n=292). Losartan equivalents were modeled on a natural log scale to accommodate observed distributions. Unexposed patients were considered to have an ARB dose = 0 Log(mg). Abbreviations: ACEi – angiotensin-converting enzyme inhibitor; ARB – angiotensin receptor blocker; MAP – mean arterial pressure; NED – norepinephrine equivalent dose; APACHE – acute physiology and chronic health evaluation score; ARDS – acute respiratory distress syndrome; RRT – renal replacement therapy; 95%CI – 95% confidence interval. | | | | | | | | |

| **Table-S17: Change in Log-Renin as Function of Losartan Dose Equivalents** | | | | | | | | |
| --- | --- | --- | --- | --- | --- | --- | --- | --- |
|  | **Model 1** | | | | **Model 2** | | | |
| Variable | Estimate | 95%CI | | p-value | Estimate | 95%CI | | p-value |
| **ARB Dose _(in Placebo)_ _log(mg)_** | **-0.59** | **-0.73** | **-0.44** | **<0.0001** | **-0.03** | **-0.11** | **0.04** | **0.40** |
| **Ang-II Treatment (vs. Placebo)** | **-0.05** | **-0.37** | **0.26** | **0.75** | **-0.55** | **-0.71** | **-0.39** | **<0.0001** |
| **ARB Dose*Treatment Interaction** | **-0.12** | **-0.44** | **0.20** | **0.46** | **0.14** | **0.03** | **0.26** | **0.0170** |
| Age _(per 10 years)_ | -0.02 | -0.06 | 0.02 | 0.87 | 0.00 | -0.06 | 0.05 | 0.91 |
| Baseline NED _log(mcg/kg/min)_ | 0.10 | -0.01 | 0.21 | 0.0648 | 0.10 | -0.02 | 0.23 | 0.11 |
| Baseline APACHE-II Score | 0.00 | -0.01 | 0.01 | 0.70 | 0.00 | -0.01 | 0.01 | 0.63 |
| Baseline MAP _(mmHg)_ |  |  |  |  | 0.01 | -0.01 | 0.02 | 0.44 |
| Baseline Albumin _(g/dL)_ |  |  |  |  | -0.03 | -0.16 | 0.09 | 0.58 |
| ARDS at Baseline |  |  |  |  | -0.06 | -0.23 | 0.11 | 0.50 |
| Chronic Kidney Disease |  |  |  |  | 0.16 | -0.01 | 0.34 | 0.0718 |
| Chronic Hypertension |  |  |  |  | -0.09 | -0.26 | 0.08 | 0.29 |
| RRT at Baseline |  |  |  |  | 0.06 | -0.10 | 0.23 | 0.46 |
| Multivariable model outputs for the change in renin at hour 3 where ARB exposure was modeled as continuous variable based on last recorded dose.. Estimates indicate the difference in Log(pg/mL) per unit change in the predictor variable. Estimates for interaction effects indicate the change in the estimate when treated with Ang-II instead of placebo. The models excluded the n=29 ACEi-exposed patients (total sample n=292). Losartan equivalents were modeled on a natural log scale to accommodate observed distributions. Unexposed patients were considered to have an ARB dose = 0 Log(mg). Abbreviations: Ang-II – angiotensin-II; ACEi – angiotensin-converting enzyme inhibitor; ARB – angiotensin receptor blocker; NED – norepinephrine equivalent dose; APACHE – acute physiology and chronic health evaluation score; ARDS – acute respiratory distress syndrome; RRT – renal replacement therapy; 95%CI – 95% confidence interval. | | | | | | | | |
